# Long-term survival trend after primary total laryngectomy for patients with locally advanced laryngeal carcinoma

**DOI:** 10.7150/jca.50404

**Published:** 2021-01-01

**Authors:** Zhongyang Lin, Hanqing Lin, Yuqing Chen, Yuanteng Xu, Xihang Chen, Hui Fan, Xiaobo Wu, Xiaoying Ke, Chang Lin

**Affiliations:** 1 Department of Otolaryngology, the First Affiliated Hospital of Fujian Medical University, Fuzhou, China.; 2 Department of Otolaryngology, Eye Ear Nose and Throat Hospital, Fudan University, Shanghai, China.

**Keywords:** laryngeal carcinoma, locally advanced, laryngectomy, conditional survival, SEER database

## Abstract

**Purpose:** To evaluate long-term survival trends after primary total laryngectomy (TL) for locally advanced laryngeal carcinoma (LC).

**Methods:** A total of 2094 patients diagnosed with locally advanced LC and underwent primary TL (1992-2011, at least 5-year follow-up) in the Surveillance, Epidemiology, and End Results (SEER) database were included in this study. Besides the traditional overall survival (OS) and cancer-specific survival (CSS) by using Kaplan-Meier curves, the 3-year conditional survival analysis was also performed to describe the long-term trends in these patients. Time-dependent multivariate competing-risk models were constructed to assess the persistent sub-distribution hazard of prognostic factors. Finally, a nomogram was developed to predict conditional cancer-specific survival.

**Results:** The curves of overall hazard and cancer-specific hazard both quickly reached the apex within the first year since TL, then decreased thereafter. In general, the CS3 steadily increased from within 5 years after TL. In the stratified CS3 analysis, the increments in patients with adverse characteristics were more pronounced. 4 years after TL, the probability of surviving the next year exceeded 90%. The time-dependent multivariate competing-risk models indicated that age and lymph node ratio (LNR) persistently contributed to the cancer-specific outcome. The nomogram based on the competing-risk model was constructed to estimate CSS probability conditional upon 3 years for advanced LC patients having survived 1, 2, and 3 years.

**Conclusion:** Most patients achieved a substantially improved survival rate after surviving a long period after primary TL. Patients diagnosed at older age and with higher LNR should receive more effective follow-up. The predictive nomogram can provide significant evidence for clinical research and practice.

## Introduction

Laryngeal carcinoma (LC) is one of the most common malignancies in head and neck worldwide 1. Squamous cell carcinoma accounts for the majority of laryngeal carcinoma 2. Approximately 43.1-44.1% of advanced LC were diagnosed in locally advanced status, which generally leads to poor prognosis (5-year survival rate between 39-55%) 3.

The treatment paradigms of laryngeal preservation derived from two landmark trials from the Veteran's Affairs Laryngeal Cancer Study (VA) and the Radiation Therapy Oncology Group (RTOG) led the shift to organ-preservation strategy as a preferred option 4, 5. However, the treatment based on primary total laryngectomy (TL) remains the irreplaceable role for its survival benefits for advanced LC in real-world clinical scenarios 3, 6.

The dynamically improvement of survival after primary treatment were recently reported in other malignancies 7, 8. Likewise, the Multidisciplinary Larynx Cancer Working Group analyzed the conditional OS for patients with advanced LC and found the mortality risk evolving over time 9. Patients with locally advanced LC might have potential reduced hazards according to the survival time after primary TL. To date, there were no existing reports primarily for disclosing the long-term survival trend of locally advanced LC after receiving primary TL. Conventional assessments of prognosis such as 5-year survival rate were relatively limited in accurate survival description, especially for patients who have survived for a long period after surgical treatment. Therefore, we aimed to investigate a large database to address this issue.

## Materials and Methods

### Study population

We conducted this retrospective study by analyzing cases from the Surveillance, Epidemiology, and End Results (SEER) database which encompasses 18 population-based cancer registries covering approximately 28% of the US population. Eligible participants who were initially diagnosed as LC between 1992 and 2011 (at least 5-year follow-up) were extracted from the SEER database by SEER*stat software (version 8.3.6).

The primary site was identified by ICD-O (International Classification of Diseases for Oncology): Glottic (C320), supraglottic (C321). The year of diagnosis was categorized as 1992-2001 and 2002-2011. Age was classified into two groups (<60 and ≥60). The cut-off point for lymph node ratio (LNR) was previously reported ranging from 0.03 to 0.14 10, 11, and in this study, we used using the “X-tile” program (Yale University, USA) to obtain the optimal point 12. Accordingly, the LNR was divided into three groups (< 0.03, ≥ 0.03, no neck dissection). Race was categorized as white and other (American Indian/AK Native and Asian/Pacific Islander). The pathological grade of tumors was categorized as high grade (poorly differentiated, undifferentiated) and low grade (well and moderately differentiated). For all enrolled patients, the clinical description of tumor status was per the 6^th^ American Joint Committee on Cancer (AJCC) staging classification.

The inclusion criteria for all cohorts were as follows: one primary malignancy only, with complete information (race, surgical procedure, cause of death), T3 or T4 stage, M0 stage, underwent total laryngectomy, follow-up more than 1 month, alive or known causes of death (COD). The all cohort were used for overall survival analysis and the construction of competing-risk regression models. The net cohort excluded cases with known COD other than LC and were exclusively adopted in the comparisons between the Kaplan-Meier curves and 3-year conditional cancer-specific survival curves.

The cancer-specific death was defined by the SEER cause-specific death classification.

### Statistical analysis

The Kaplan-Meier (K-M) curves using log-rank test were adopted for each variable to assess the association with OS and CSS by using the log-rank test. The K-M analysis was conducted by using all cohort for OS, and the net cohort (excluding other COD) for CSS, respectively. Further, the results of K-M analysis were compared with the corresponding CS3. Variables revealed significant relevance to both OS and CSS were selected as covariates in the multivariate competing-risk models.

Condition survival (CS), as an alternative concept, demonstrate dynamic trends of survival probability according to the duration of follow-up^13^, ^14^. The CS3 estimates represented the probability of survival for an additional 3 years, provided the patient survived for 1, 2, 3, 4, or 5 years. CS3 = S(X + 3)/S(X): For example, the 3-year CS among patients who had survived for 1 year from the date of surgery was calculated by dividing the 4-year survival rate by the 1-year survival rate. In the present study, the OS and CSS were assessed conditional upon 3 years survival.

The time-dependent multivariate competing-risk models based on the Fine and Gray method were conducted for patients having survived 1, 2, 3, and 4 years since TL besides baseline, evaluating the long-term cancer-specific contribution of prognostic factors among patients who survived for a period after surgery.

Finally, a predictive nomogram 15 for conditional survival estimation was generated according to the multiple competing-risk model. The accuracy of the nomogram was tested by the Harrell's concordance index (C-index).

In the present study, *p* < 0.05 (two-sided) was considered significant in all tests. The analyses above were performed using the X-tile program and the R software (Version 3.6.0 R Foundation).

## Results

### Baseline characteristics

The baseline characteristics of enrolled cohorts were summarized in Table 1. A total of 2094 patients diagnosed as locally advanced LC were included in this study. There were 585 patients died of causes other than LC excluded from all cohort, and the remaining 1509 patients were defined as the net cohort for the CSS analysis. Compared with the net cohort, patients who died of causes other than LC were significantly more prone to be diagnosed during 1992-2002, older, better lymph node status, and absence of adjuvant therapy.

### Traditional survival and conditional survival

**Figure 1:**
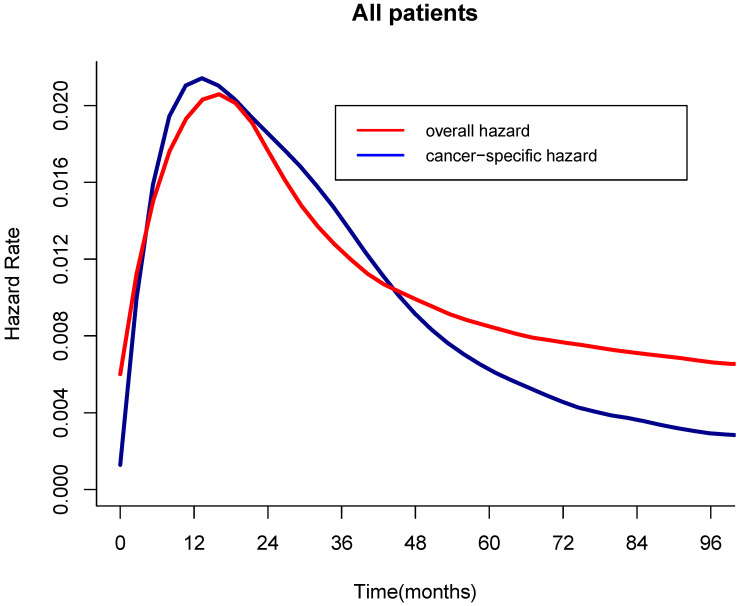
** Hazard curves of overall and cancer-specific death over time.** a, Overall hazard; b, Cancer-specific hazard.

**Figure 2:**
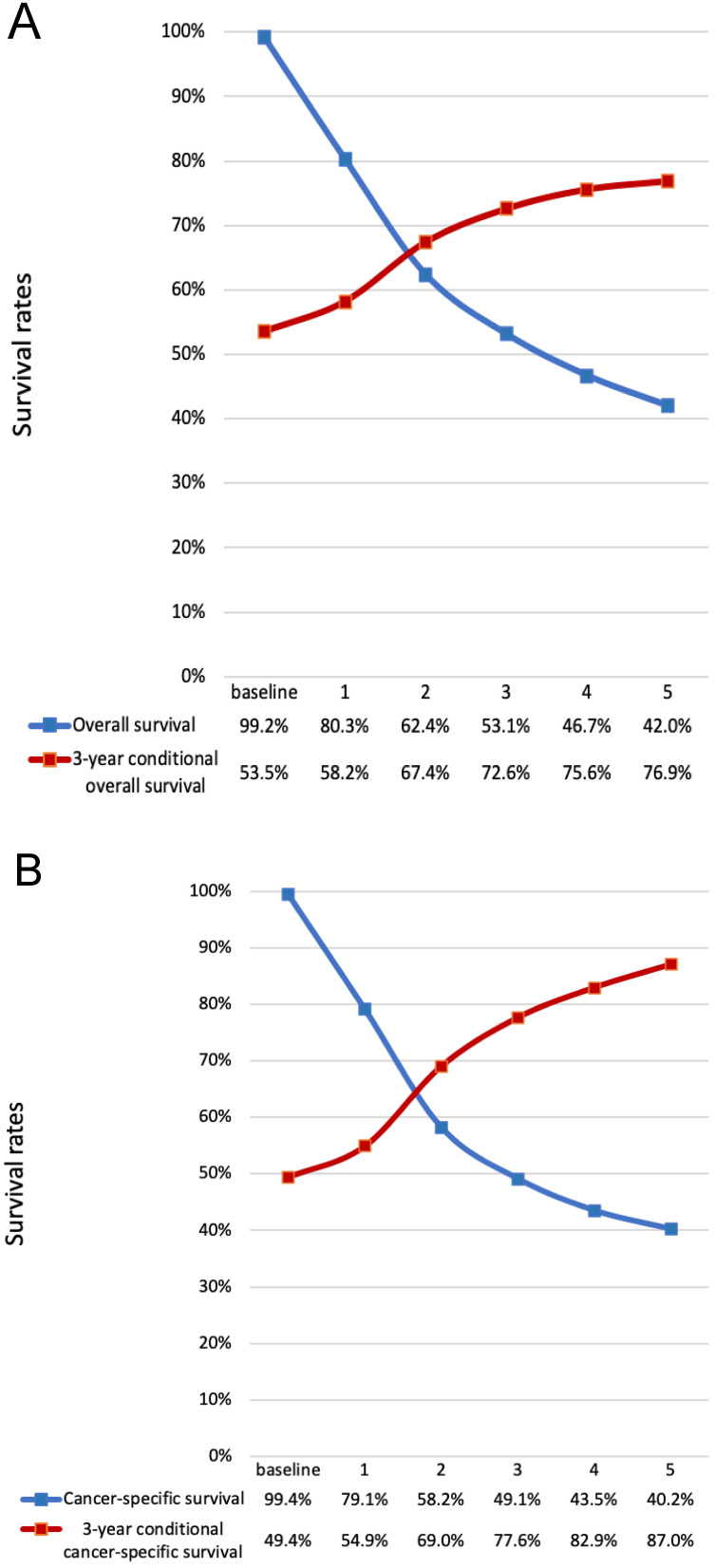
** Comparison of traditional K-M curves with CS3 for all cohort and the net cohort.** a, Traditional overall survival and 3-year conditional OS for all cohort. b, Traditional CSS and 3-year conditional CSS for the net cohort. CSS, cancer-specific survival; CS3, 3-year conditional survival; K-M, Kaplan-Meier; OS, overall survival.

For the all cohort, the median and meantime of follow-up was 41 months and 64 months (range one month to 298 months). During the follow-up, 1595 (76.17%) patients died, and of these, 1010(63.32%) patients died of LC. According to the Figure 1, the hazard curves of revealed that the risks of overall and cancer-specific death were not constant, which increased swiftly and reached the peak within the first year since surgery then declined thereafter. As can be seen from the Figure 2, the postoperative trends of K-M curves and the corresponding CS3 for both OS and CSS were compared respectively. With 5-year elapsing after TL, the OS declined from 99.2% to 44.0%. At odds with the trend of traditional K-M curve, the overall 3CS steadily increased from 53.5% at baseline to 76.90% at the 5^th^ year since surgery. After 2-year survival from TL, the rate was 67.0%, compared with the 5-year OS of 42.0%. The K-M curve and 3CS also indicated resemble trends for the CSS.

**Figure 3:**
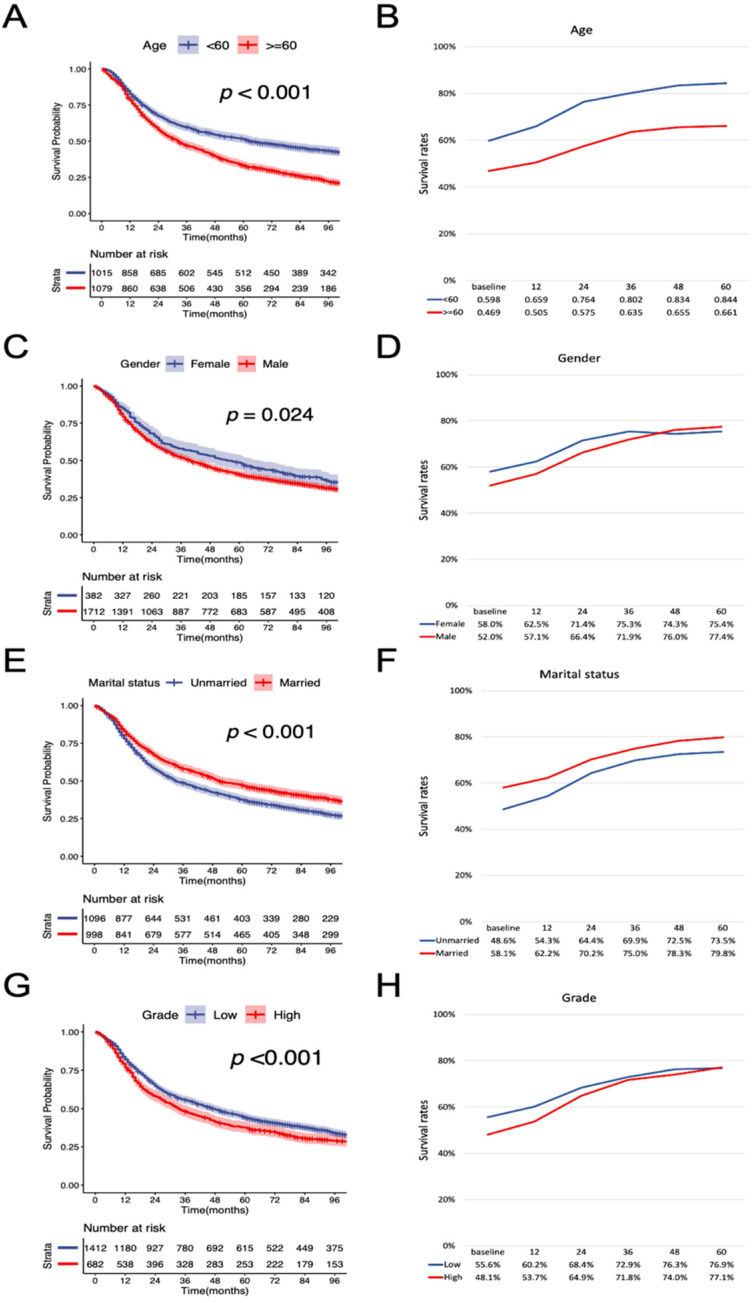

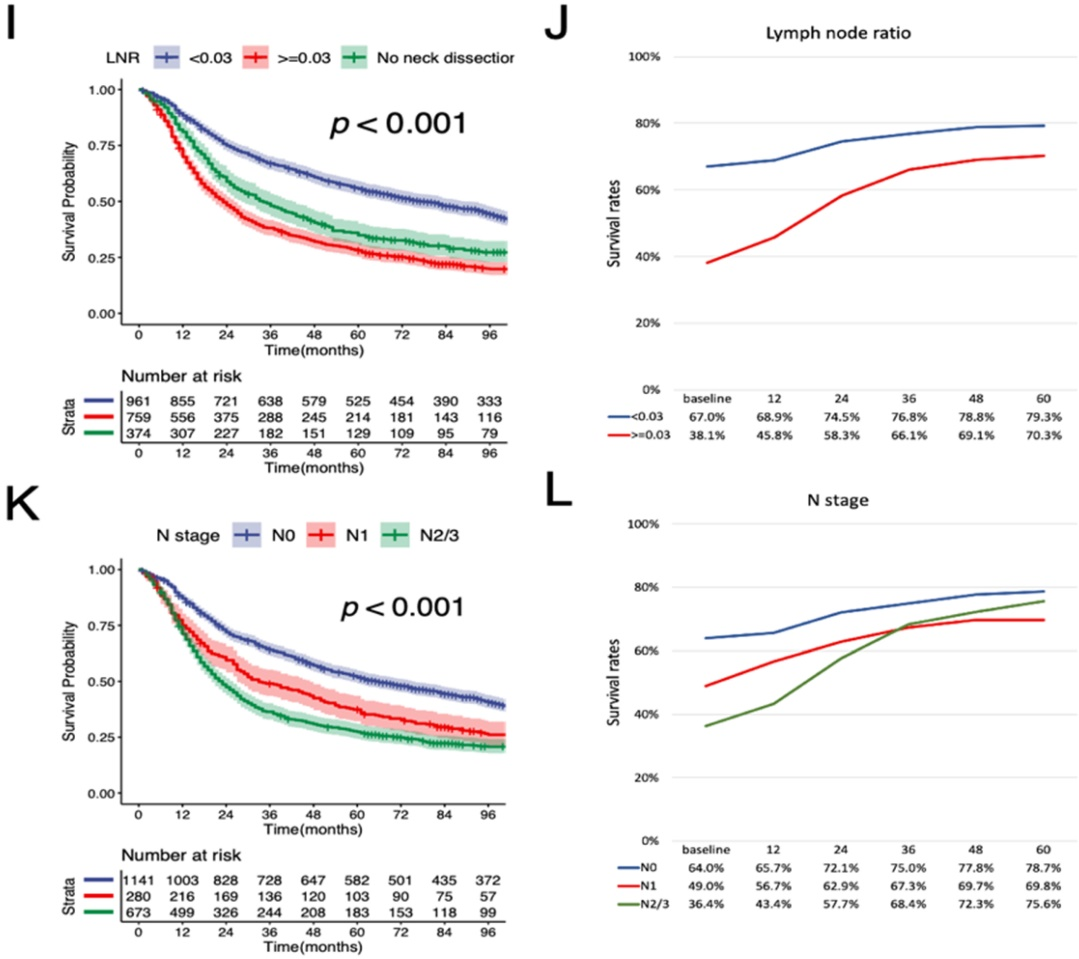
** Stratified comparison of the OS with the 3-year conditional OS in age, gender, marital status, pathological grade, LNR, and N stage.** Patients were stratified according to (A, B) age, (C, D) gender, (E, F), marital status, (G, H) pathological grade, (I, J) LNR, and (K, L) N stage. OS, cancer-specific survival; LNR, lymph node ratio.

**Figure 4:**
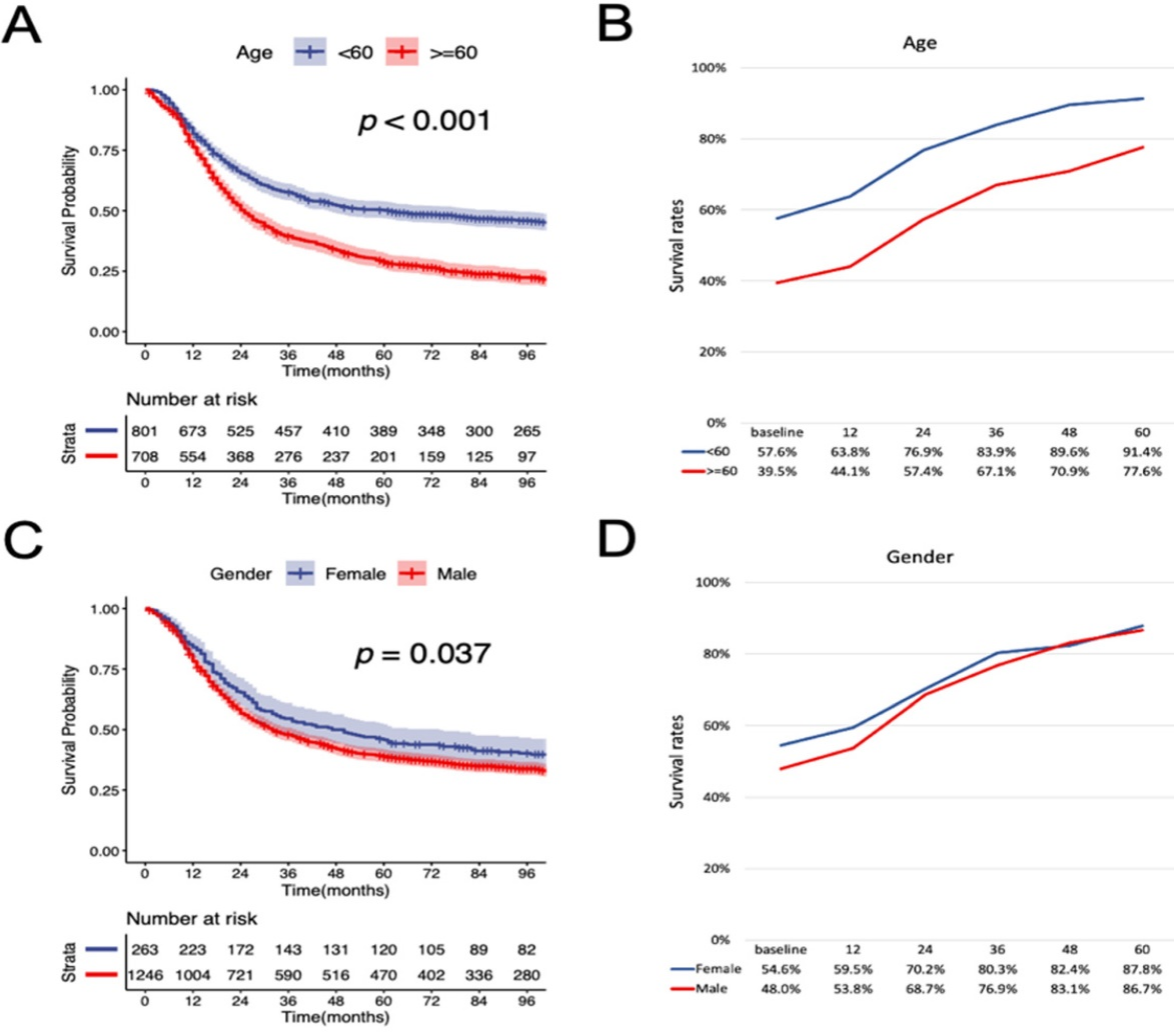

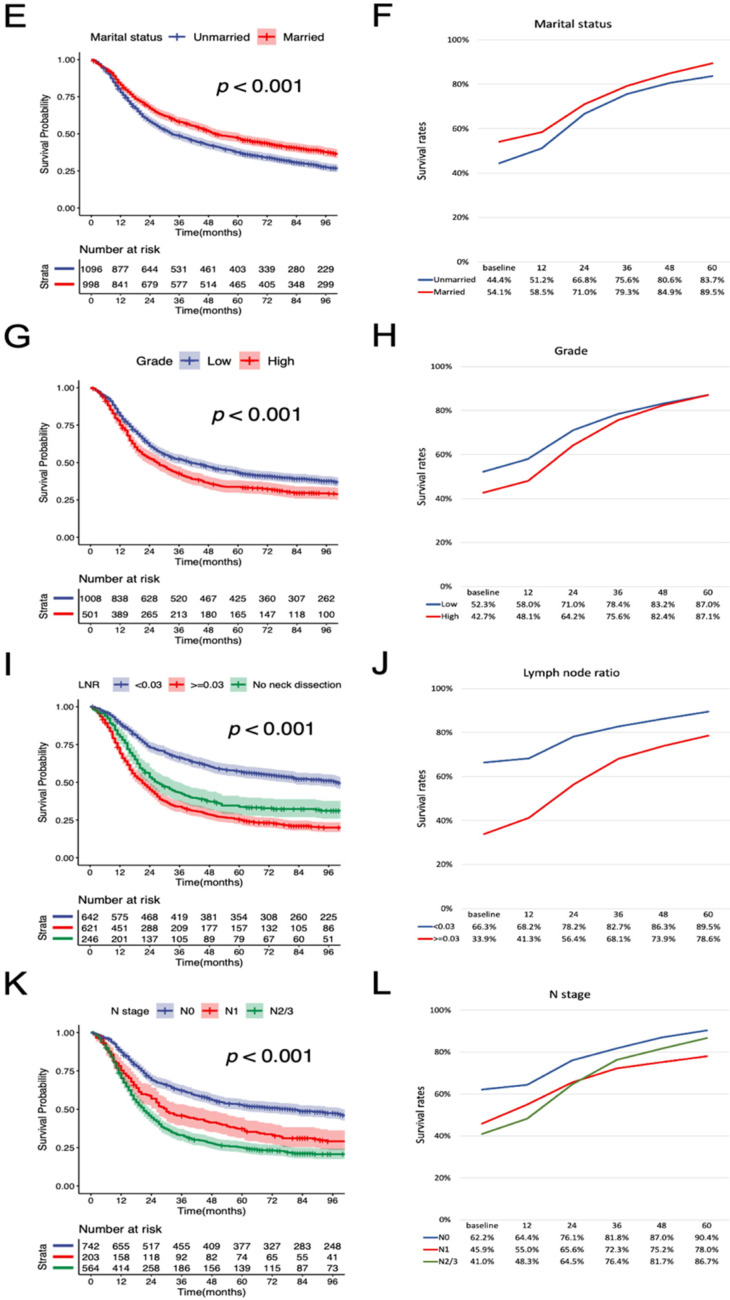
** Stratified comparison of the CSS with the 3-year conditional CSS in age, gender, marital status, pathological grade, LNR, and N stage.** Patients were stratified according to (A, B) age, (C, D) gender, (E, F), marital status, (G, H) pathological grade, (I, J) LNR, and (K, L) N stage. CSS, cancer-specific survival; LNR, lymph node ratio.

According to the K-M curves, year of diagnosis, age, pathological grade, LNR, N stage were significantly related to both OS and CSS (Figure 3 and Figure 4), the results of other variables were presented in the supplementary material. It is clear that the increments of CS3 were more pronounced in those patients who were initially diagnosed with adverse characteristics. The range between CS3 estimation of most variables correspondingly exhibited narrowing trends with the prolongation of time after TL. For instance, for patients with LNR≥ 0.03, the 3-year conditional OS was 38.1% at baseline and reached 70.3% (+32.2%) after five years, and the 3-year conditional CSS increased by 44.7% during this period. By contrast, patients with LNR<0.03, the 3-year conditional OS and CSS had an increase of 12.3% and 23.2% respectively.

**Figure 5:**
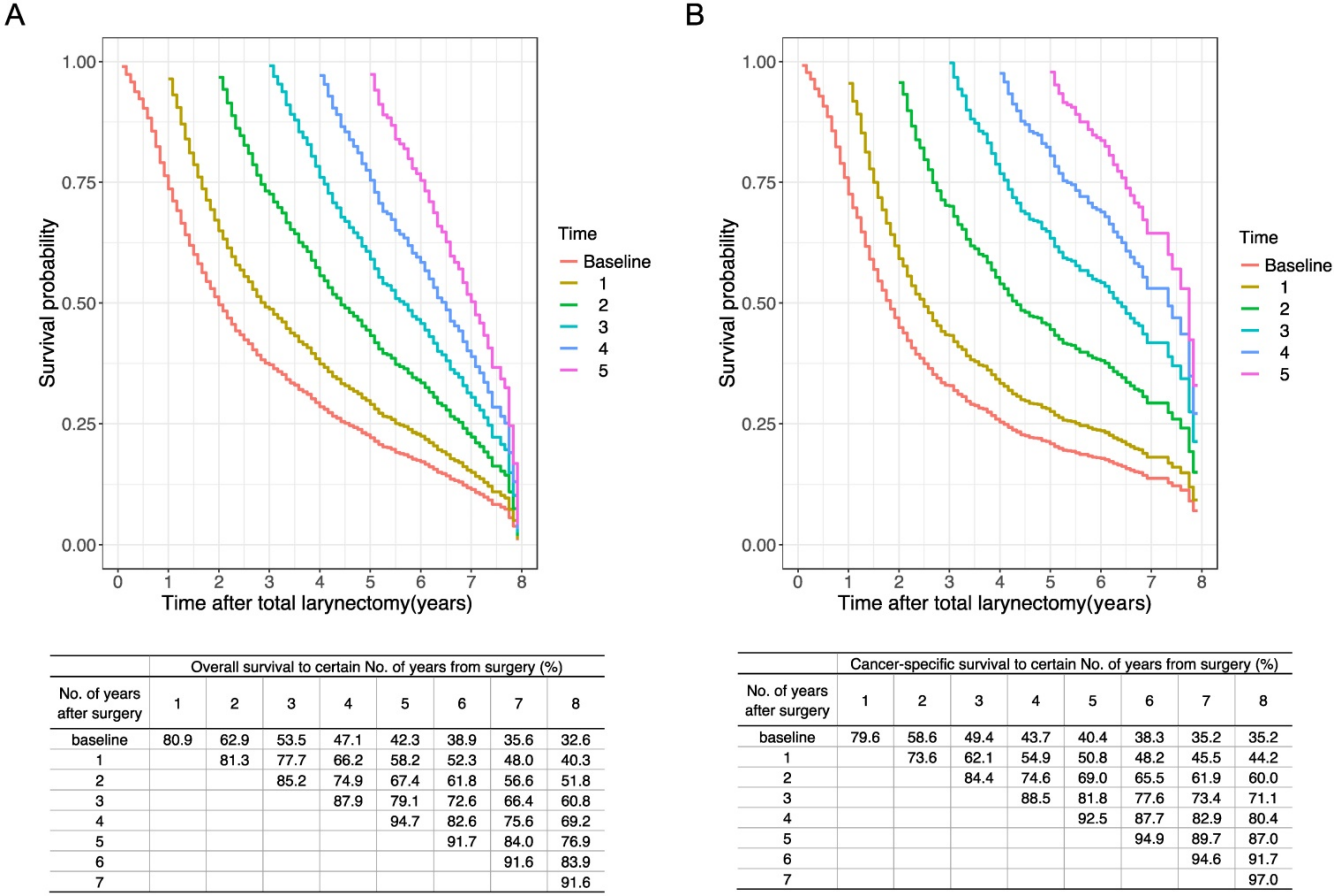
** Conditional OS and CSS estimations in 1-year interval.** Kaplan-Meier curves of (A) OS and (B) CSS after surgery at 1-year interval for all patients. The table inside the figure shows the corresponding survival probability of surviving a certain number of years after surgery according to surviving time since surgery. CSS, cancer-specific survival; OS, overall survival.

Furthermore, we estimated the conditional OS and CSS based on the number of years patients had already survived (Figure 5). The 5-year conditional OS improved from 42.3% at the time of surgery to 52.3%, 56.6%, and 60.8% for patients who had survived 1, 2, 3 years respectively after surgery. For 5-year conditional CSS, the probability increased by 30.7% after 3-year surviving.

### Time-dependent multivariate models

The multivariate competing-risk regression models based on the Fine-Gray method were constructed to evaluate the contribution of prognostic factors at baseline and subsequent 4 years at 1-year interval (Table 2). Age, gender, marital status, pathological grade, LNR, and N stage were identified as prognostic factors by the K-M curves. Taking competing-risk events into account, age, gender, marital status, LNR, and N stage were significantly associated with cancer-specific survival at baseline of follow-up. Whereas, marital status lost cancer-specific prognostic significance after 1-year survival, followed by gender and N stage at the beginning of the 3rd and 4^th^ year. The constantly high sub-distribution hazard illuminated that age and LNR showed persistent contribution of prognosis in the five time-dependent multivariate models.

### Development of predictive nomogram

**Figure 6:**
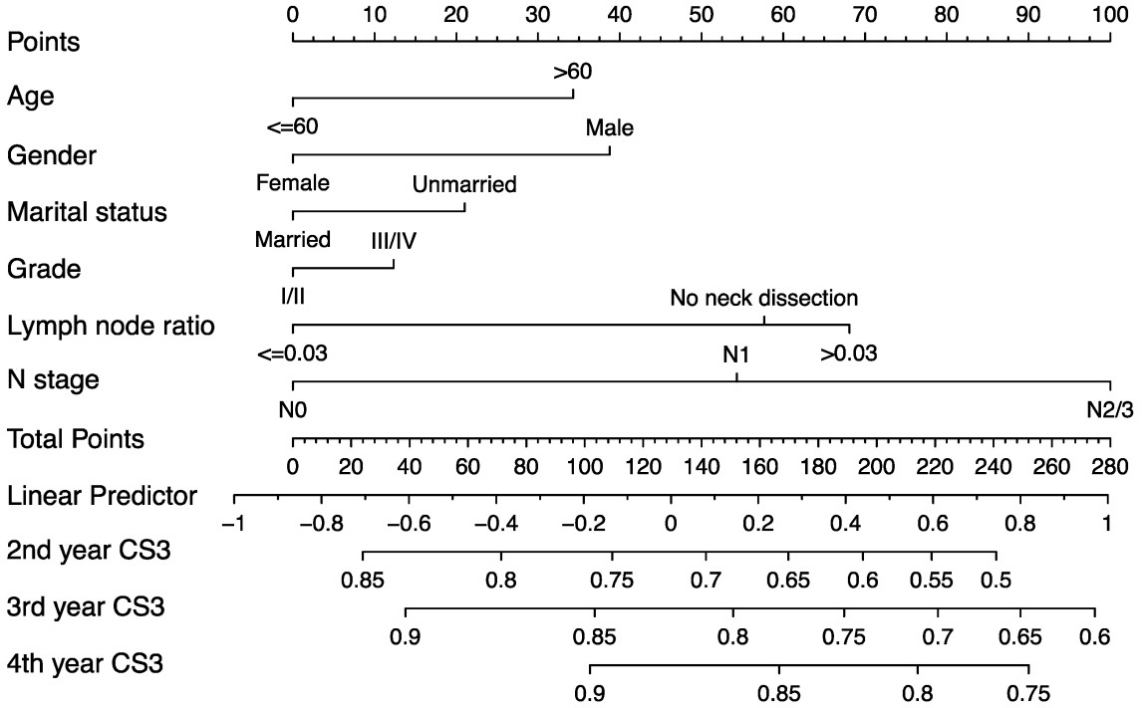
Predictive nomogram of individualized 3-year conditional cancer-specific survival estimation for patients having survived 1, 2, and 3 years.

The predictive nomogram was developed based on the multivariate competing-risk regression model (Figure 6). The 3-year conditional CSS at the beginning of 2^nd^, 3^rd^ and 4^th^ year could be estimated by using the nomogram. The C-index of the model was 0.651 (95% confidence interval: 0.635-0.667).

## Discussion

The treatment option between laryngeal preservation and laryngectomy of locally advanced LC has been extensively controversial for quite long time. Despite the matching survival outcome in selected candidates from the VA and RTOG trials, the survival of advanced LC decreased over the past few decades. Several studies attributed the worsening observation to the generalization of the organ preservation strategy 6, 16. The primary TL still yields the favored survival results in the population-based studies 6, 17, 18 and continued to be the standard treatment. However, the long-term survival trend of advanced LC after primary TL remained ambiguous, and there are a lack of relevant researches focusing on this issue. With this regard, our study firstly applied stratified CS analysis and time-dependent multivariate models, offering unprecedented insights into the long-term survival evaluation of locally advanced LC after primary TL. The methodology has been routinely utilized in other primary malignancies 13, 19, 20.

In the present study, we found that the hazard of death was not monotonic over time. For both OS and CSS, the hazard soared within the first year since TL, then decreased thereafter. The trend of the hazard curve indicated that patients had a high risk of death after TL within a short period, but the survivors could have a relatively low hazard or even cure with prolonged follow-up. In another way, the early deaths of patients with high hazards complied with the “natural selection” of low-risk patients, thereby optimized the prognosis of surviving patients gradually.

We further assessed the long-term trend of the OS and CSS by using the concept of CS3 in these patients. For OS and CSS, CS3 exhibited a steadily upward trend, compared to the traditional decreasing trend of K-M curves. More substantial improvements in CS3 were observed for patients with initially adverse characteristics. As follow-up continued, the range between the significantly favorable and adverse in most characteristics narrowed. The results indicate that locally advanced LC patients have a considerable reduction in both overall and cancer-specific hazard with long survival after surgery. For patients having survived four years from TL, the probability of surviving the next year exceeding 90% (Figure 5), which means an extreme encouragement for these patients.

During the follow-up period time, 585 (27.9%) competing events were observed. In general, previous studies constructed traditional Cox regression models for either OS or CSS, which failed to evaluate the effect on cumulative incidence. As such, we constructed the competing-risk regression model based on the Fine-Gray method that manages these two mutually exclusive outcomes. Moreover, it is far from enough for survival estimation to conventionally evaluate the effect of prognostic factors merely at the baseline of the follow-up. We conducted multivariate regression models based on subsequent 4-year time point after surgery to assess the long-term contribution of prognostic factors. The time-dependent models indicated the significance of continuous assessment in prognostic evaluation, which was generally employed in the various CS analysis studies 14, 20, 21. Age, gender, marital status, LNR, and N stage showed significant influence on prognosis at the beginning of follow-up. According to the sub-distribution hazard ratio in time-dependent models, only age and LNR consistently exhibited influences on long-term cancer-specific survival. It has been reported that age was associated with conditional overall survival in advanced LC during the follow-up after diagnosis 9. The existing literature deduced that the reason for this observation is the higher pack-years of smoking 22, and the predominant proportion of male gender also supported this speculation in part. The cumulation of tobacco could hardly be eliminated even after primary TL. It was recognized by several studies that the tumor invasive ability manifests largely in the form of lymph node ratio 11, 23, 24. Patients with a higher LNR had inferior long-term survival than those with low LNR. The prognostic predicting significance of LNR on patients with laryngeal cancer calculated at baseline has been described recently 25, 26. This surrogate mathematical marker was also included in the nomogram recently proposed by Zhu et al. and facilitates risk stratification for the decision of adjuvant treatment 27. Nevertheless, the prognostic influence of LNR has not been validated in the long-term follow-up by time-dependent regression models. In our results, LNR revealed superior long-term predictive ability than the TNM staging system, and the sub-distribution hazard was maintained a relatively substantial level. It seems that the conventional AJCC staging classification is prone to be a more anatomically descriptive parameter, but not a determinant parameter for the prognostic prediction. It should be kept in mind that the extra-nodal extension (ENE) included in the latest version was not recorded in the SEER database for patients diagnosed before 2018. Whether the latest N staging classification combined with ENE would be prominent in the prediction of survival outcome is worth expecting. Prospective researches were warranted to seek the optimal prognostic parameters for these patients.

In the era of precision medicine, developing postoperative surveillance strategies only according to the survival estimation at baseline is insufficient to some extent. Due to the inadequate evidence, the current guidelines are also lack of specific individualized follow-up recommendations for patients underwent primary TL. The follow-up frequency should be adjusted for dynamic changes of survival. We generated a predictive nomogram based on the multivariate competing-risk model for clinical practice. The good predictive ability of nomogram enables clinicians and patients to estimate the conditional cancer-specific survival based on the time patients have survived. The estimation of the nomogram can serve as a reliable reference for cancer research, clinical consultations, and follow-up strategies.

There were several inevitable limitations in our study. Firstly, the detail information about radiation and chemotherapy in the SEER database is incomplete. Secondly, crucial parameters were not provided by the SEER database such as HPV status, smoking, and extra-nodal extension for laryngeal cancer, which were outlined of prognostic value in previous studies 28, 29. Thirdly, further external validation of the predictive nomogram is required for general use. Despite the limitations, it is the strength of adequate cases and valid follow-up from the SEER database that allows us to conduct this comprehensive analysis for long-term survival evaluation and fills the blankness of this relevant field.

## Conclusion

In conclusion, this study firstly investigated the SEER database to assess the dynamic survival trend after primary TL in locally advanced LC. We conducted comprehensive survival analysis for these patients. Most of them achieved a substantially improved survival rate after surviving a long period after surgery. Patients diagnosed at an older age and with higher LNR need receive more effective follow-up. Additionally, we developed a nomogram to predictive CSS survival conditional upon 3 year for patients having survived 1, 2, and 3 years.

## Supplementary Material

Supplementary file.

## Figures and Tables

**Table 1:** Clinicopathological characteristics of enrolled cohorts

Characteristic	All cohort	Net cohort (excluding other COD)	Died of other causes	p#
Total	2094	1509	585	
**Year of diagnosis**				<0.001
1992-2001	818 (39.06%)	518 (34.33%)	300 (51.28%)	
2002-2011	1276 (60.94%)	991 (65.67%)	285 (48.72%)	
**Age**				<0.001
<60	1015 (48.47%)	801 (53.08%)	214 (36.58%)	
≥60	1079 (51.53%)	708 (46.92%)	371 (63.42%)	
**Gender**				0.144
Female	382 (18.24%)	263 (17.43%)	119 (20.34%)	
Male	1712 (81.76%)	1246 (82.57%)	466 (79.66%)	
**Race**				0.093
White	1604 (76.6%)	1141 (75.61%)	463 (79.15%)	
Other	490 (23.4%)	368 (24.39%)	122 (20.85%)	
**Marital status**				0.860
Unmarried	1096 (52.34%)	777 (51.49%)	319 (54.53%)	
Married	998 (47.66%)	732 (48.51%)	266 (45.47%)	
**Primary site**				0.385
Glottic	953 (45.51%)	684 (45.33%)	269 (45.98%)	
Supraglottic	1141 (54.49%)	825 (54.67%)	316 (54.02%)	
**Grade**				0.213
Low	1412 (67.43%)	1008 (66.8%)	404 (69.06%)	
High	682 (32.57%)	501 (33.2%)	181 (30.94%)	
**LNR**				<0.001
<0.03	961 (45.89%)	642 (42.54%)	319 (54.53%)	
≥0.03	759 (36.25%)	621 (41.15%)	138 (23.59%)	
Not examined	374 (17.86%)	246 (16.3%)	128 (21.88%)	
**T stage**				0.552
T3	579 (27.65%)	411 (27.24%)	168 (28.72%)	
T4	1515 (72.35%)	1098 (72.76%)	417 (71.28%)	
**N stage**				<0.001
N0	1141 (54.49%)	742 (49.17%)	399 (68.21%)	
N1	280 (13.37%)	203 (13.45%)	77 (13.16%)	
N2/3	673 (32.14%)	564 (37.38%)	109 (18.63%)	
**Radiotherapy**				<0.001
No	601 (28.7%)	385 (25.51%)	216 (36.92%)	
Yes	1493 (71.3%)	1124 (74.49%)	369 (63.08%)	
**Chemotherapy**				<0.001
No	1615 (77.13%)	1114 (73.82%)	501 (85.64%)	
Yes	479 (22.87%)	395 (26.18%)	84 (14.36%)	

COD: causes of death; LNR: lymph node ratio. #Derived from χ^2^ test for categorical variables.

**Table 2:** Time-dependent multivariate competing-risk regression models

Prognostic factors	Baseline			≥ 1 year			≥ 2 years			≥ 3 years			≥ 4 years		
	SHR	95% CI	*P* value	SHR	95% CI	*p* value	SHR	95% CI	*p* value	SHR	95% CI	*p* value	SHR	95% CI	*p* value
**Age**															
<60	Ref			Ref			Ref			Ref			Ref		
≥60	1.26	1.12-1.42	<0.001*	1.34	1.16-1.54	<0.001*	1.36	1.13-1.65	0.001*	1.38	1.09-1.77	0.009*	1.67	1.24-2.23	0.001*
**Gender**															
Female	Ref			Ref			Ref			Ref			Ref		
Male	1.3	1.11-1.52	0.001*	1.29	1.07-1.55	0.007*	1.16	0.91-1.49	0.229	1.17	0.86-1.59	0.321	1.09	0.75-1.57	0.661
**Marital status**															
Unmarried	Ref			Ref			Ref			Ref			Ref		
Married	0.87	0.77-0.98	0.019*	0.88	0.76-1.01	0.069	0.91	0.75-1.1	0.308	0.95	0.75-1.21	0.681	0.94	0.7-1.27	0.705
**Grade**															
Low	Ref			Ref			Ref			Ref			Ref		
High	1.09	0.96-1.23	0.187	1.08	0.94-1.26	0.284	1	0.81-1.22	0.963	1.03	0.79-1.35	0.818	0.84	0.6-1.18	0.322
**LNR**															
<0.03	Ref			Ref			Ref			Ref			Ref		
≥0.03	1.58	1.26-1.97	<0.001*	1.53	1.17-2	0.002*	1.67	1.15-2.41	0.007*	1.91	1.09-3.34	0.024*	2.45	1.18-5.08	0.016*
Not examined	1.47	1.23-1.75	<0.001*	1.44	1.18-1.77	<0.001*	1.3	0.97-1.73	0.078	0.96	0.66-1.41	0.843	0.81	0.49-1.32	0.396
**N stage**															
N0	Ref			Ref			Ref			Ref			Ref		
N1	1.44	1.14-1.81	0.002*	1.39	1.06-1.83	0.016*	1.41	0.96-2.05	0.077	1.05	0.62-1.76	0.867	0.99	0.52-1.89	0.969
N2/3	1.95	1.54-2.47	<0.001*	1.88	1.42-2.5	<0.001*	1.66	1.12-2.46	0.012*	1.08	0.6-1.97	0.795	0.8	0.37-1.75	0.576

LNR: lymph node ratio; SHR: sub-distribution hazard ratio.

## Abbreviations

LC: Laryngeal carcinoma
VA: Veteran's Affairs Laryngeal Cancer Study
RTOG: Radiation Therapy Oncology Group
TL: total laryngectomy
SEER: Surveillance, Epidemiology, and End Results
COD: causes of death
K-M: Kaplan-Meier
OS: overall survival
CSS: cancer-specific survival
AJCC: American Joint Committee on Cancer
CS: conditional survival
CS3: 3-year conditional survival
