# Supplementary figures and images for: Long-term survival trend after primary total laryngectomy for patients with locally advanced laryngeal carcinoma
Source: J Cancer. 2021 Jan 1;12(4):1220–30. doi: 10.7150/jca.50404 (PMC7797640; doi:10.7150/jca.50404)

Overall survival

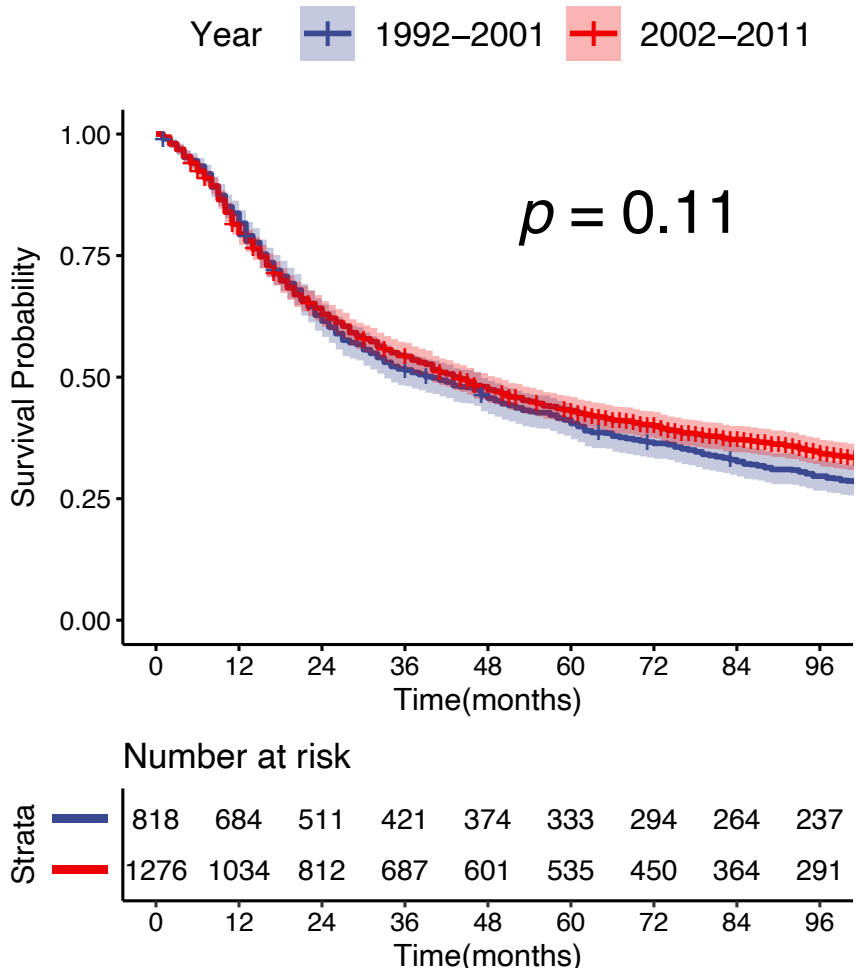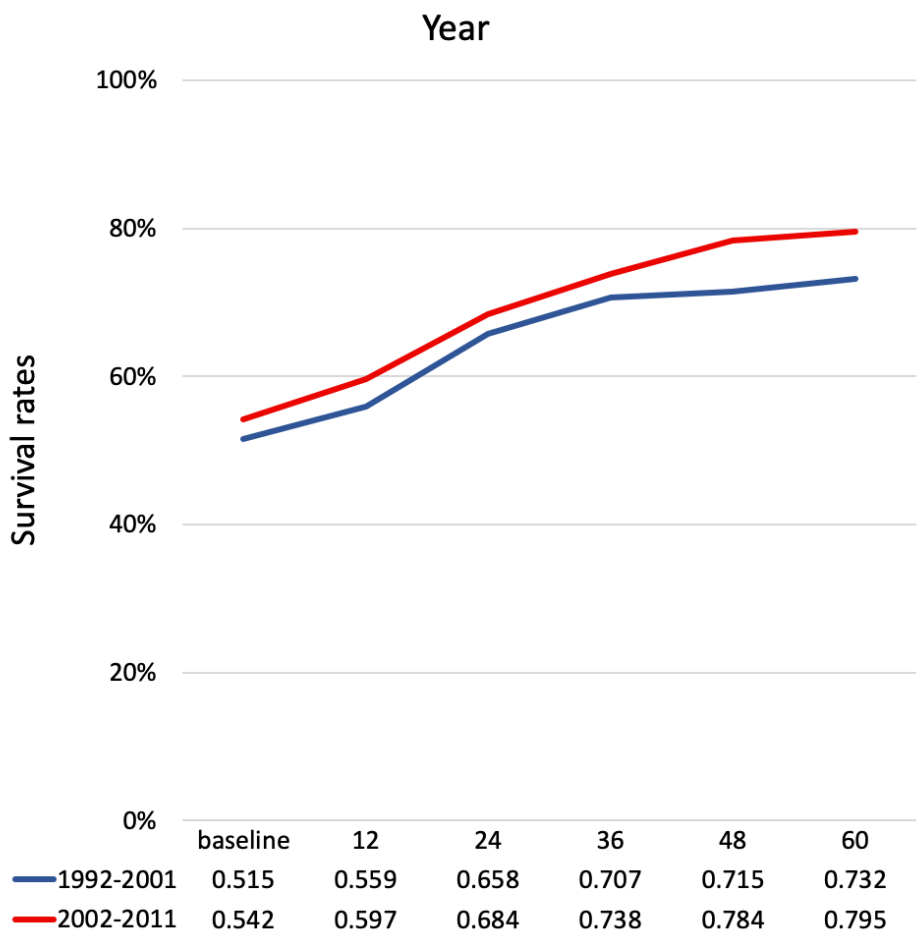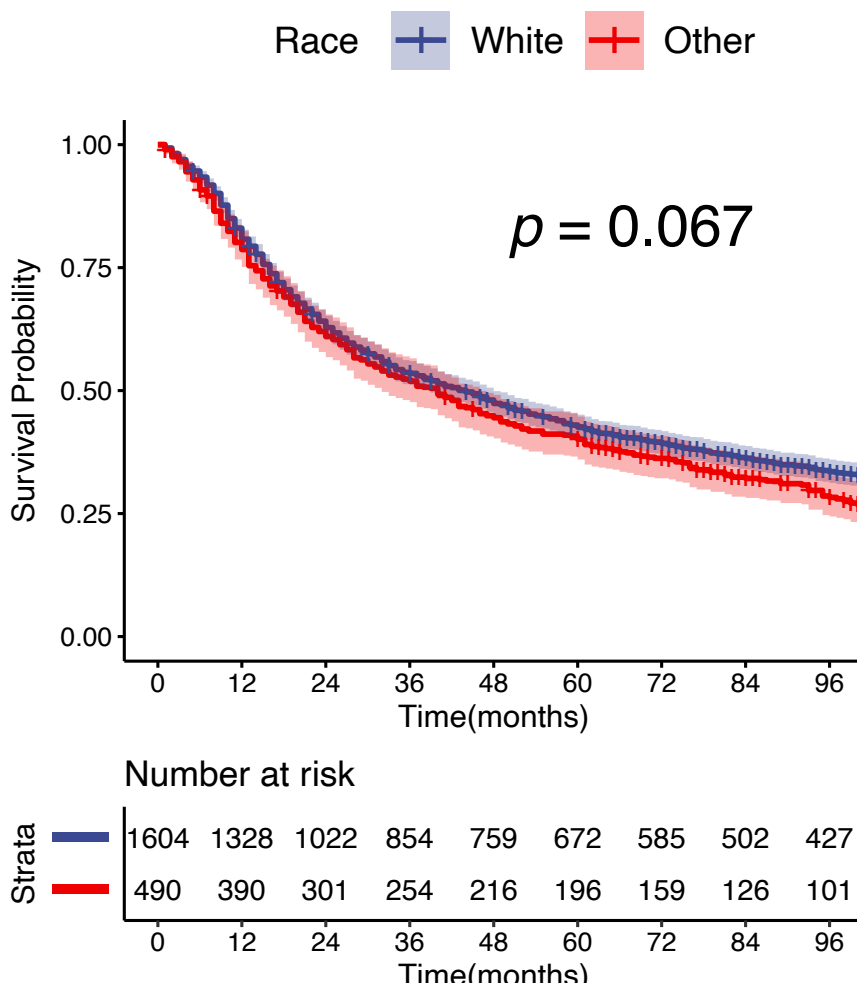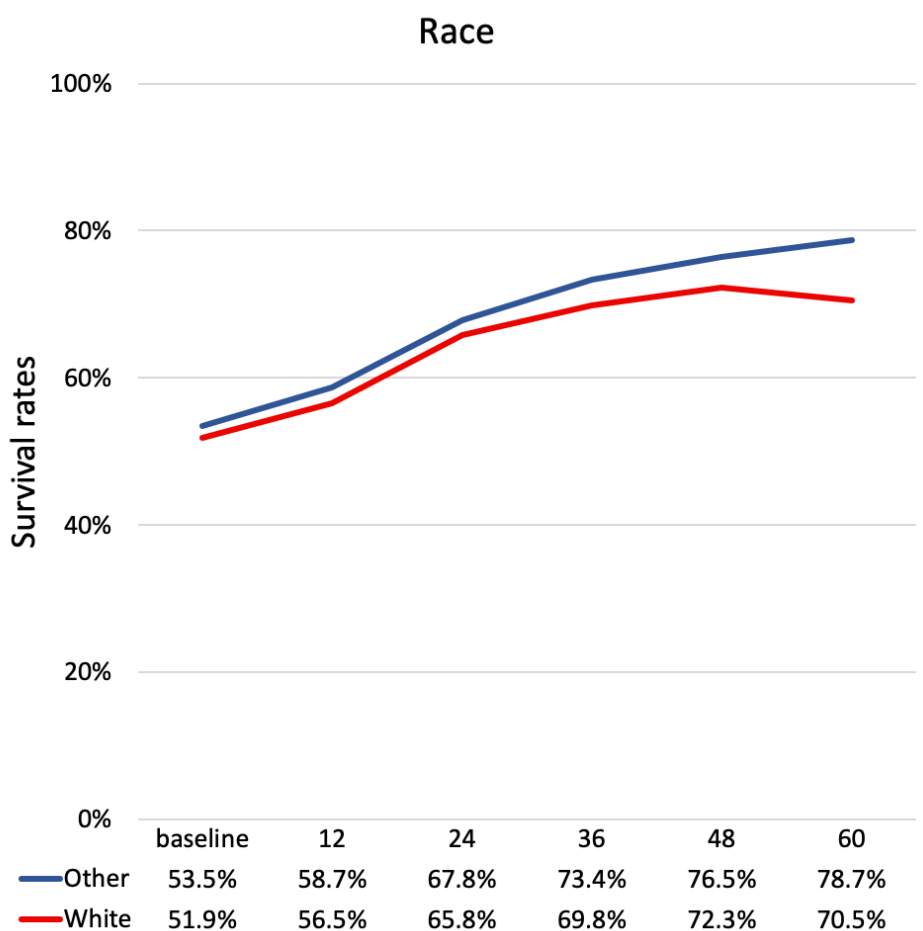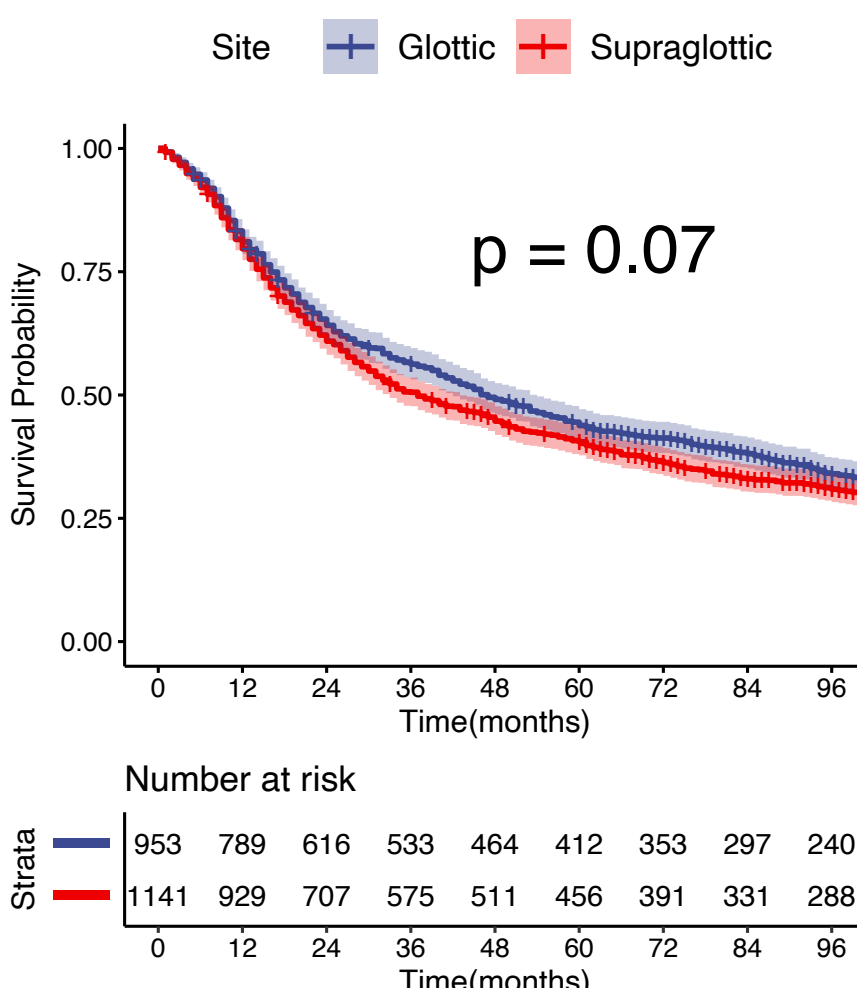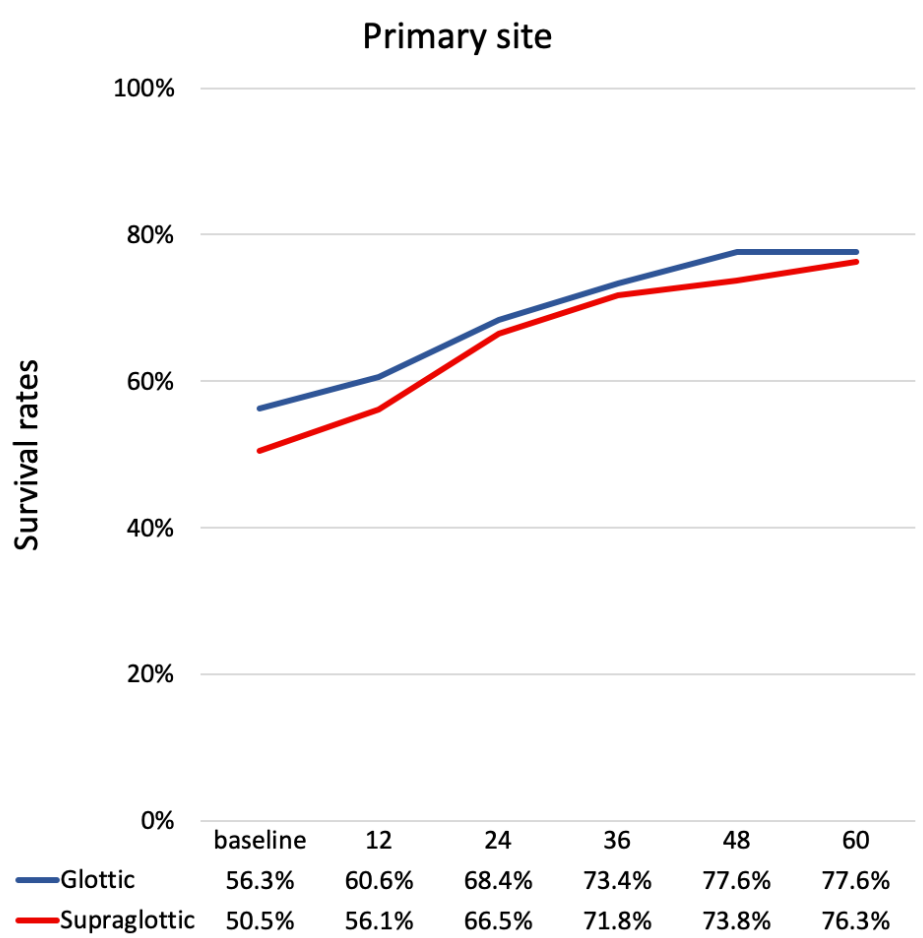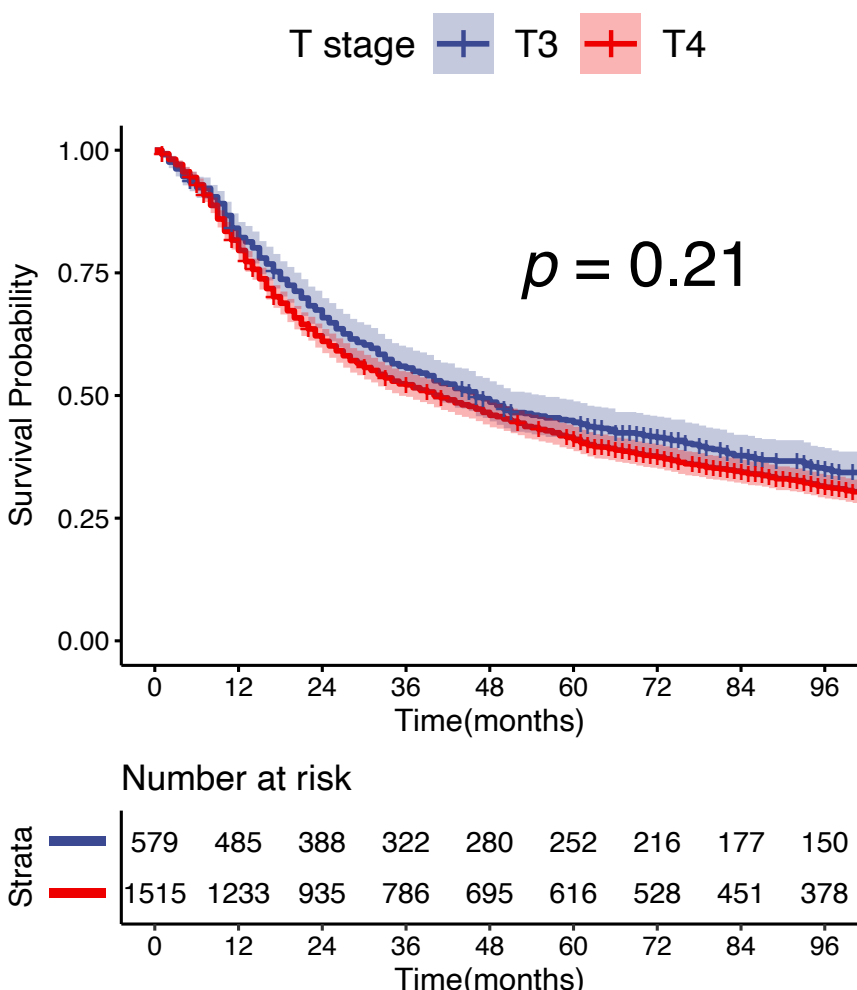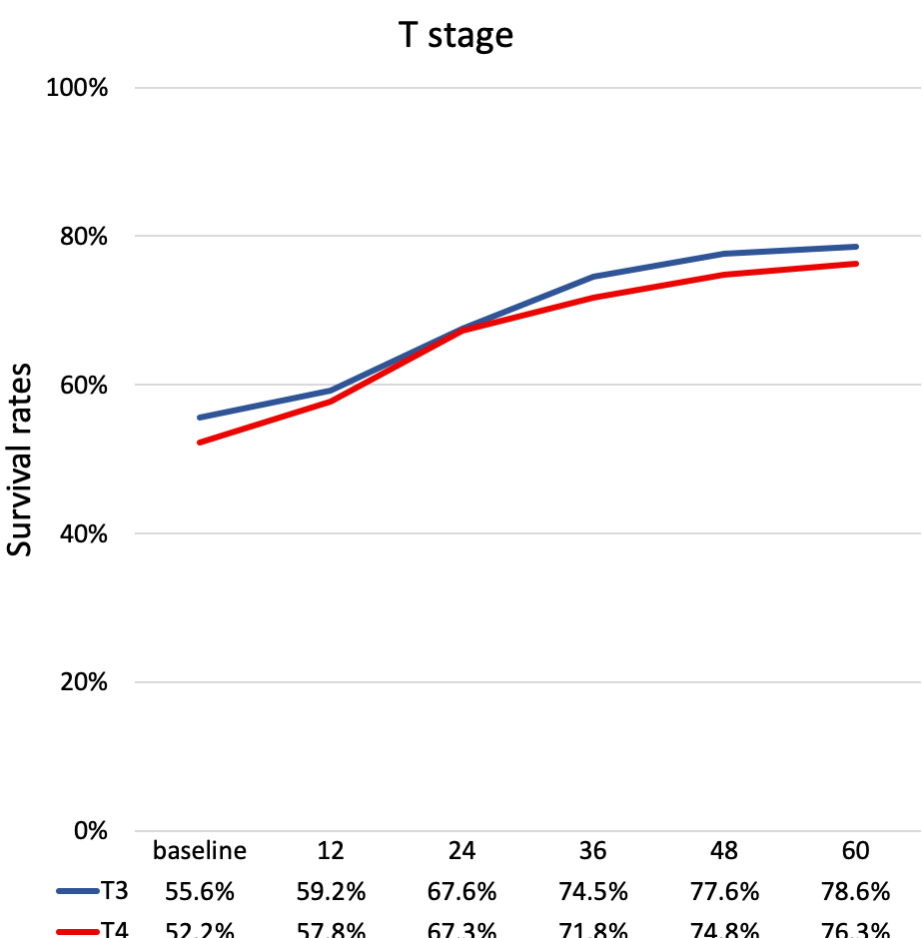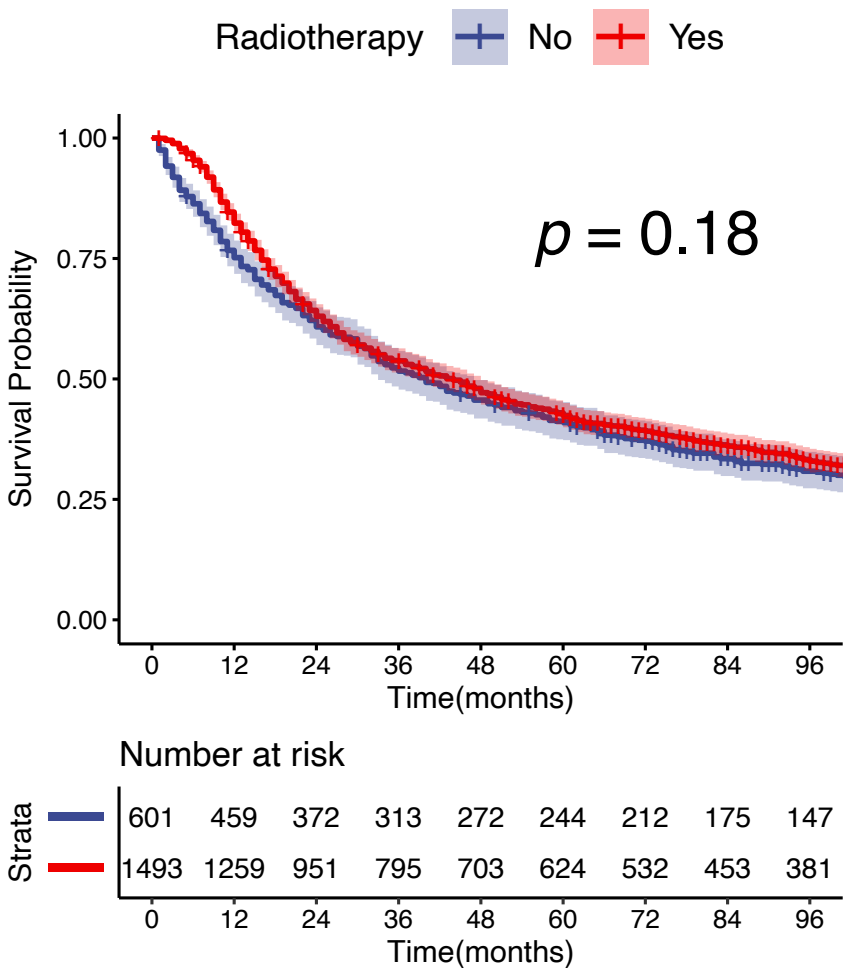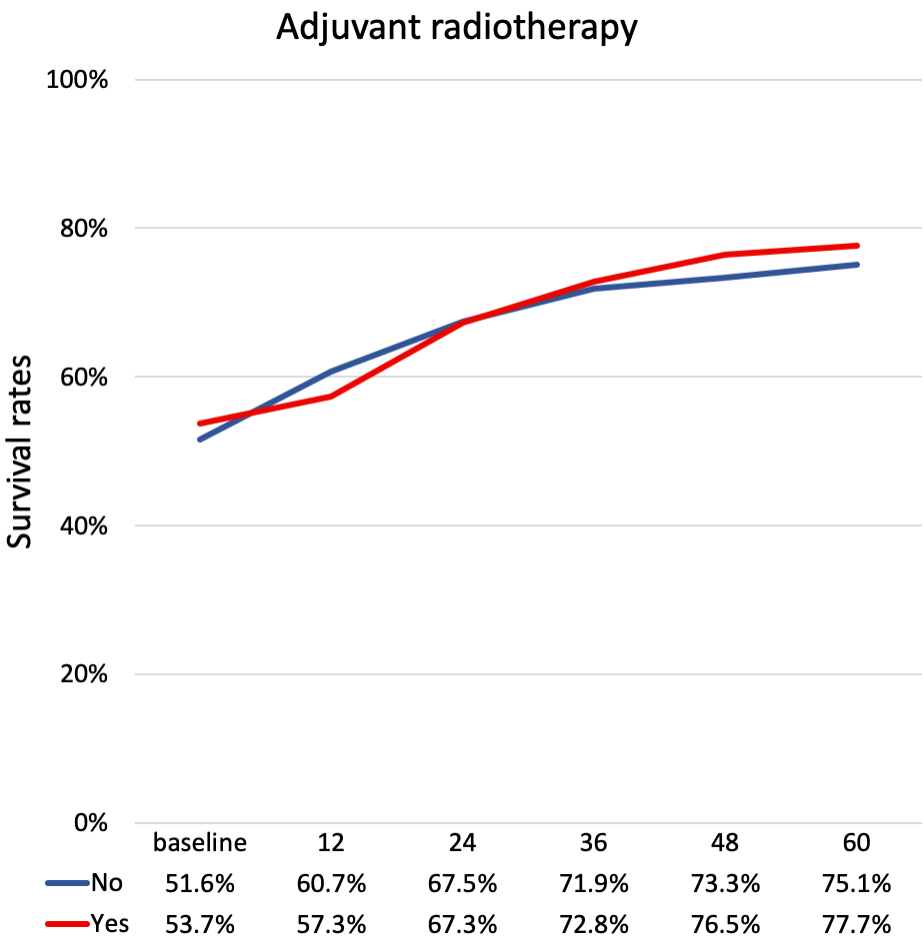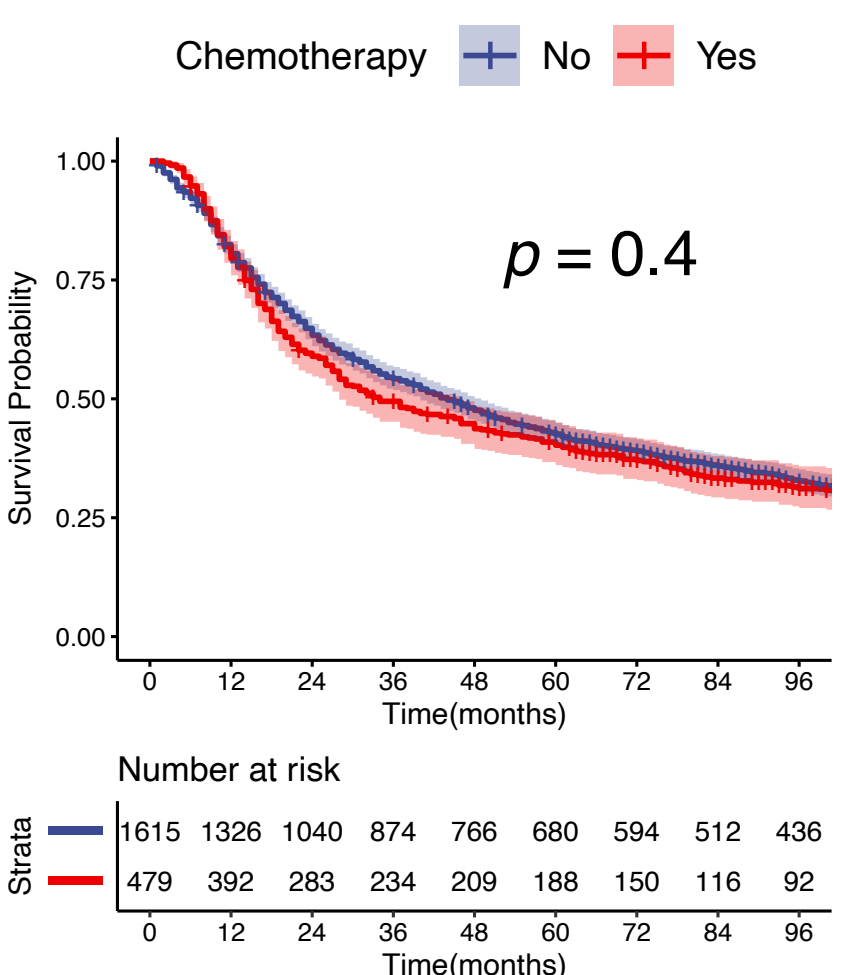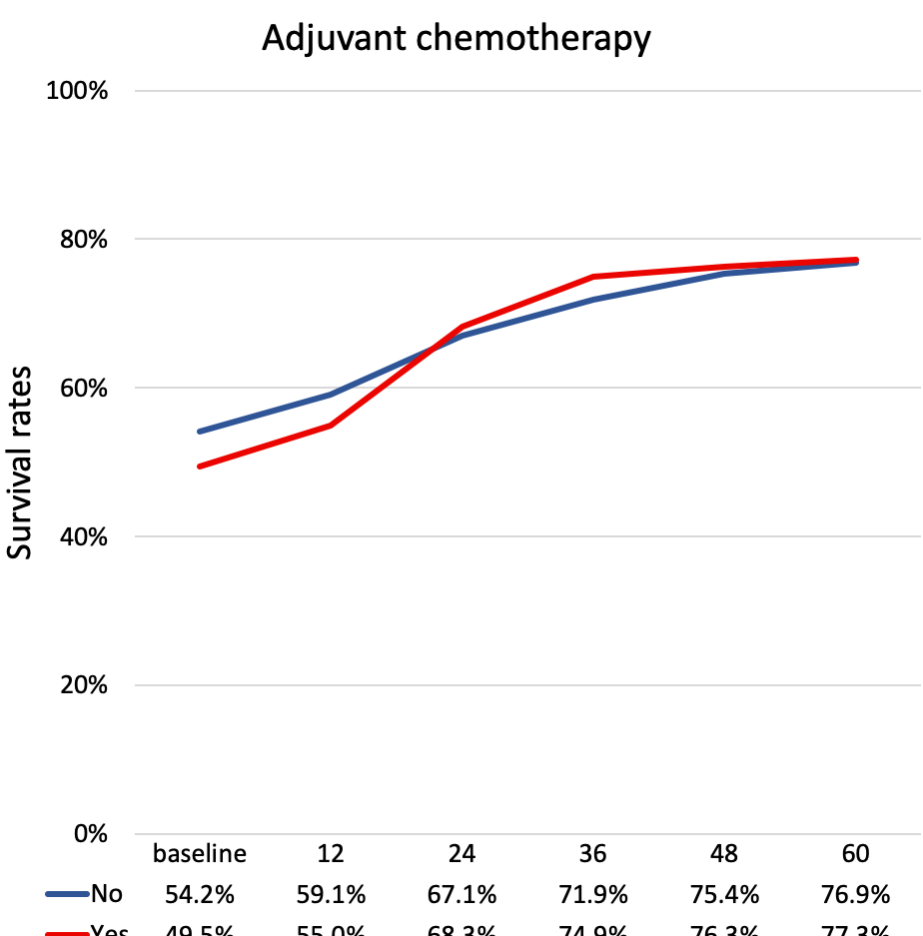

Cancer-specific survival

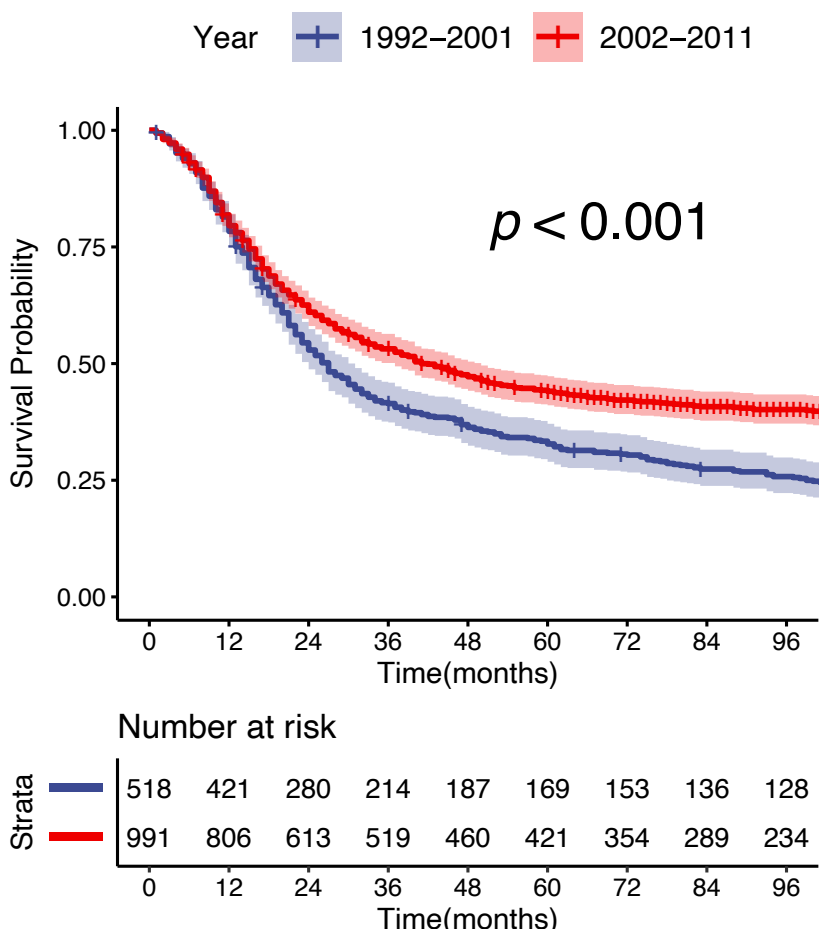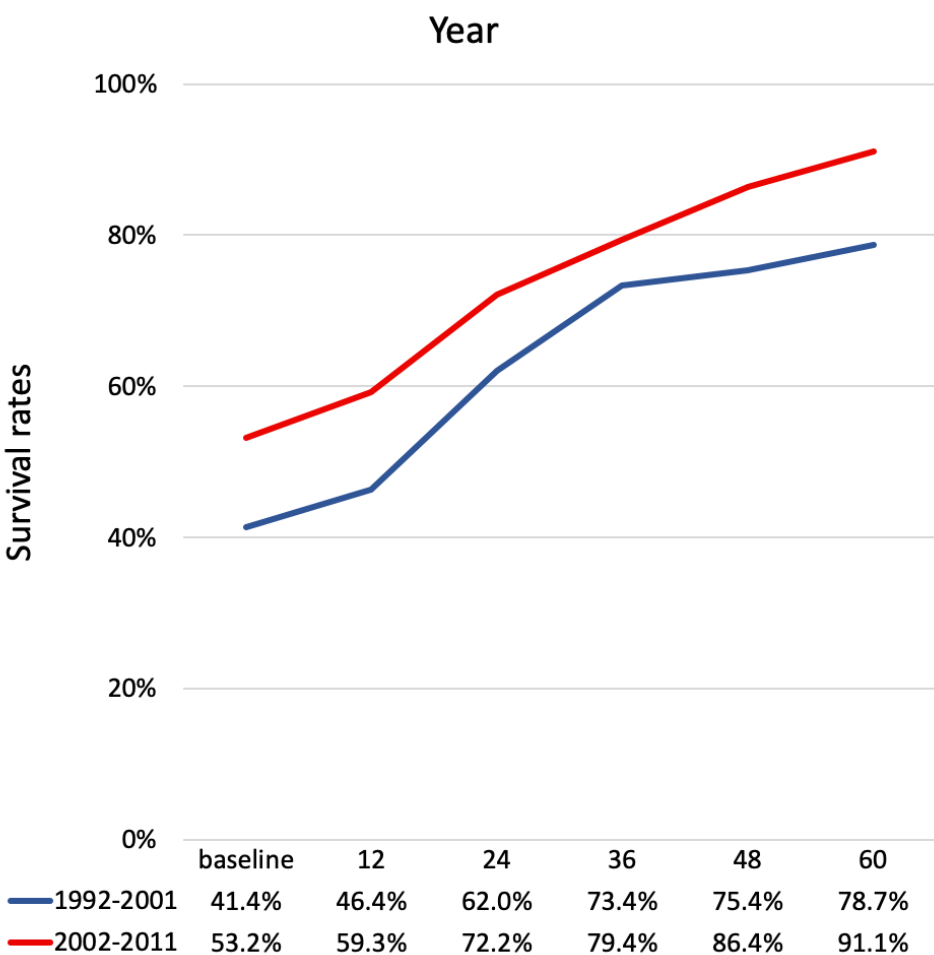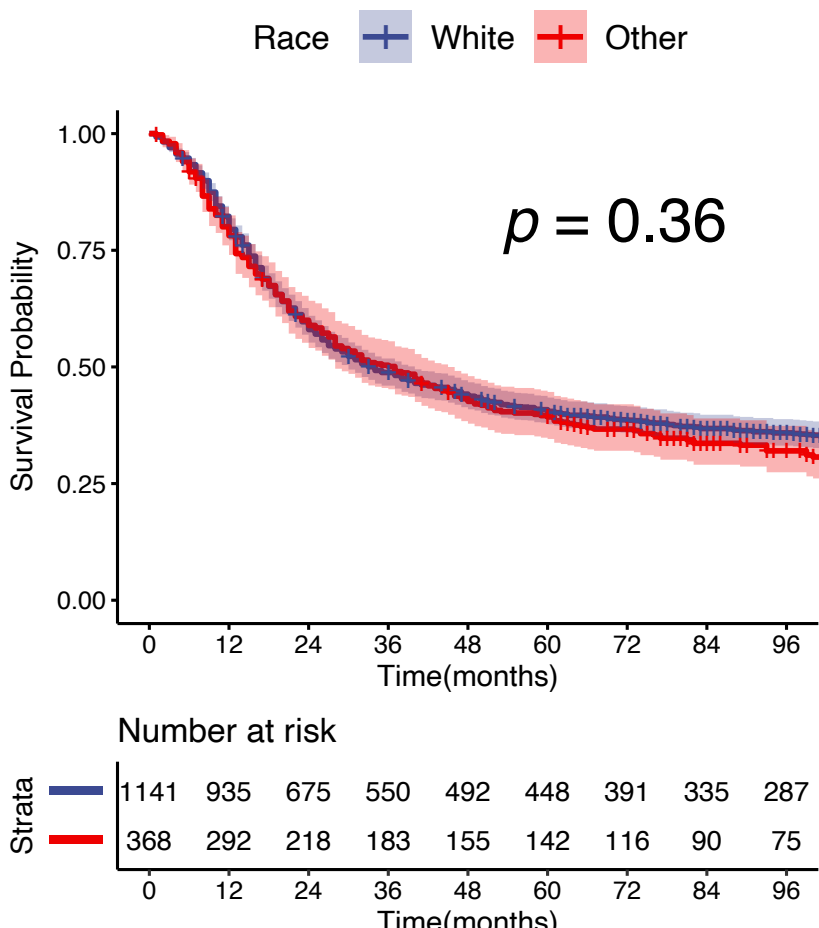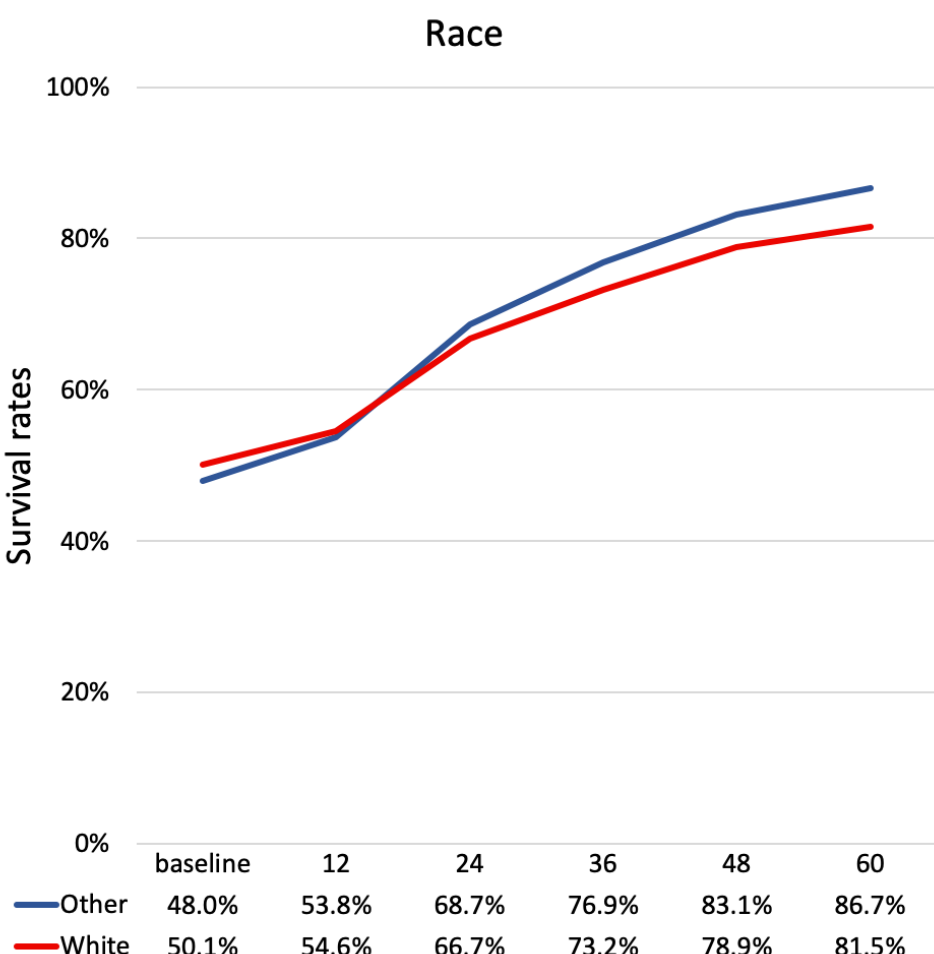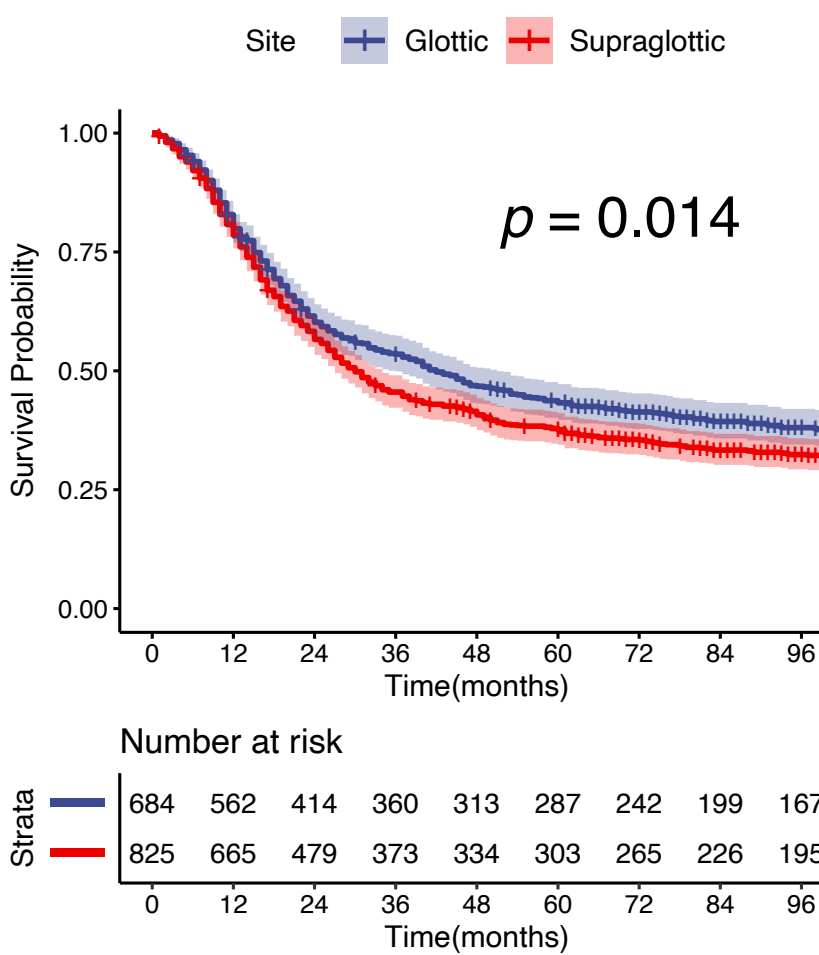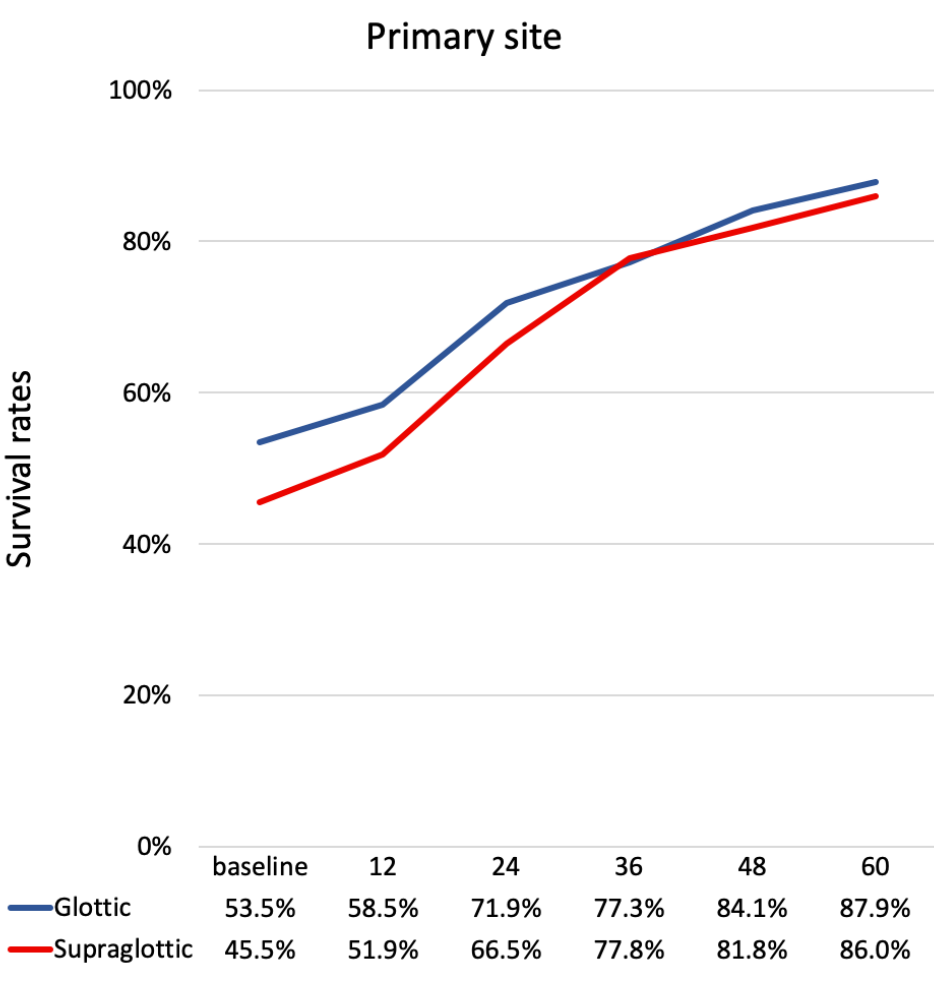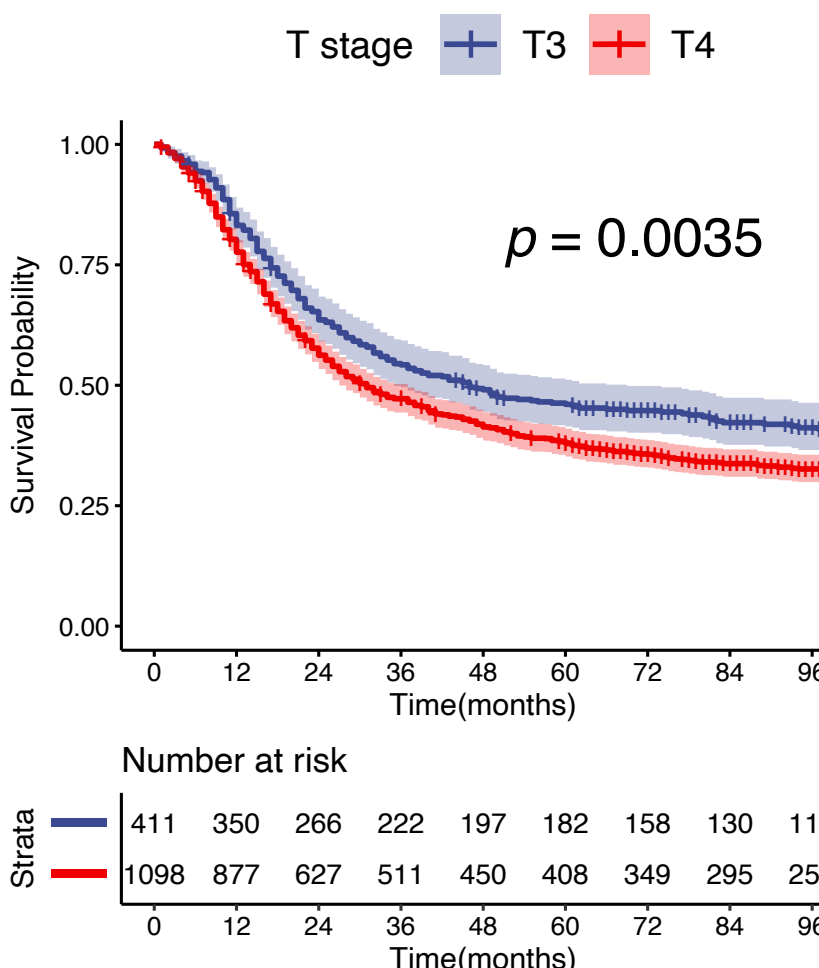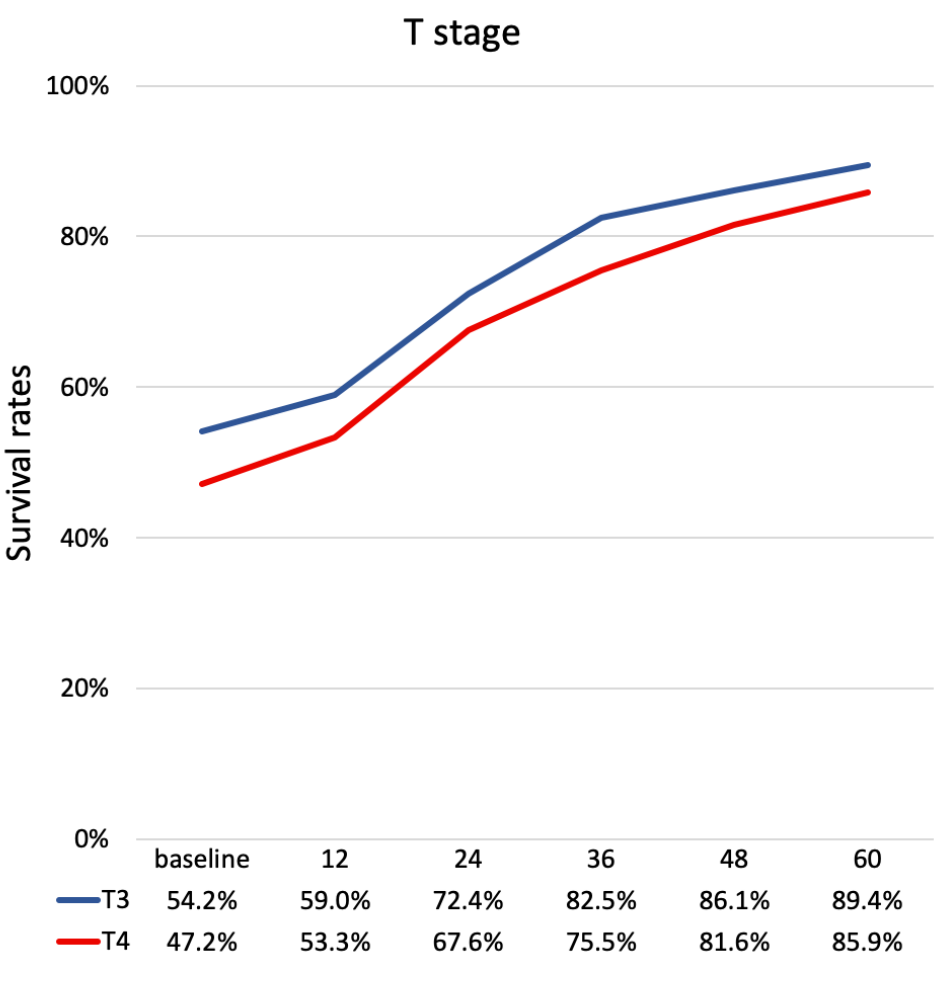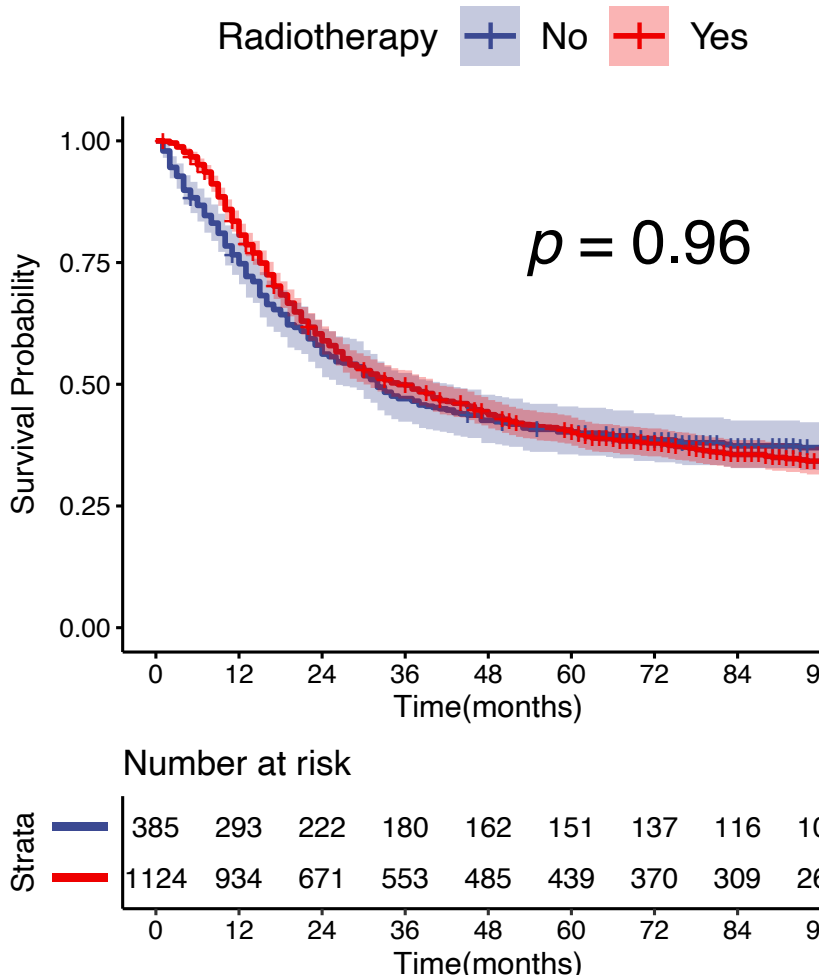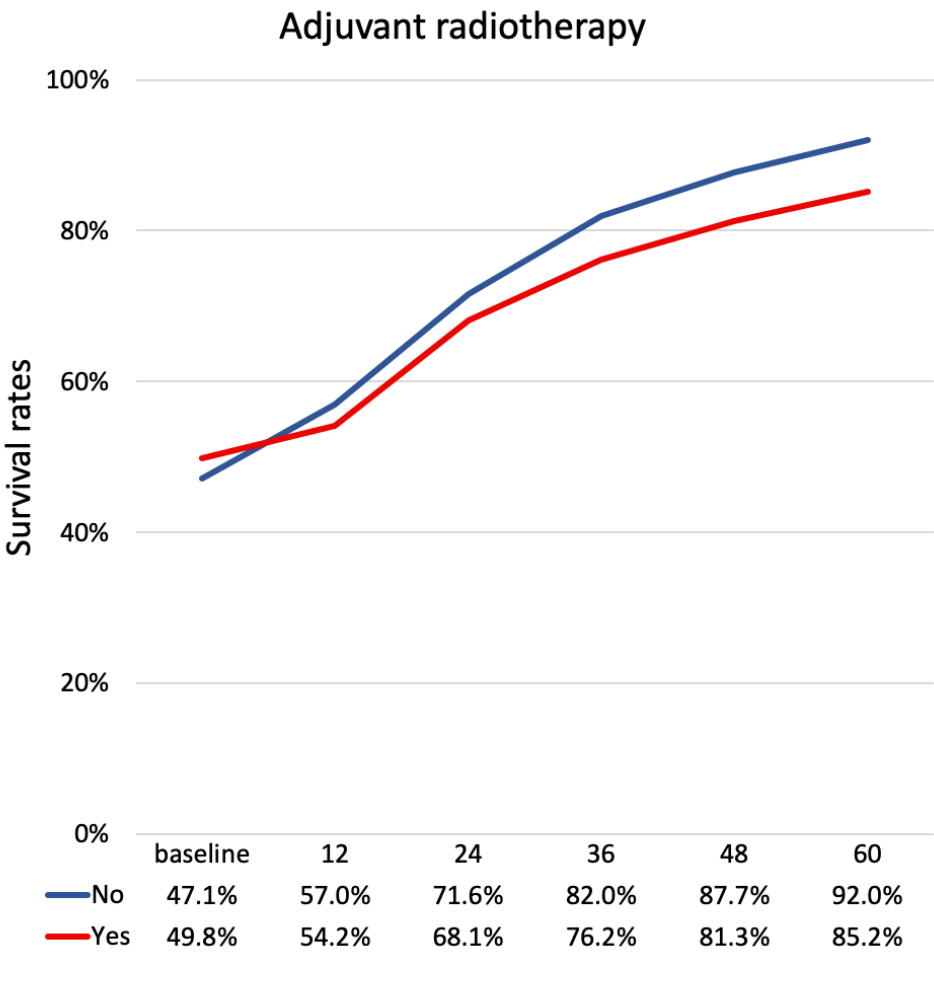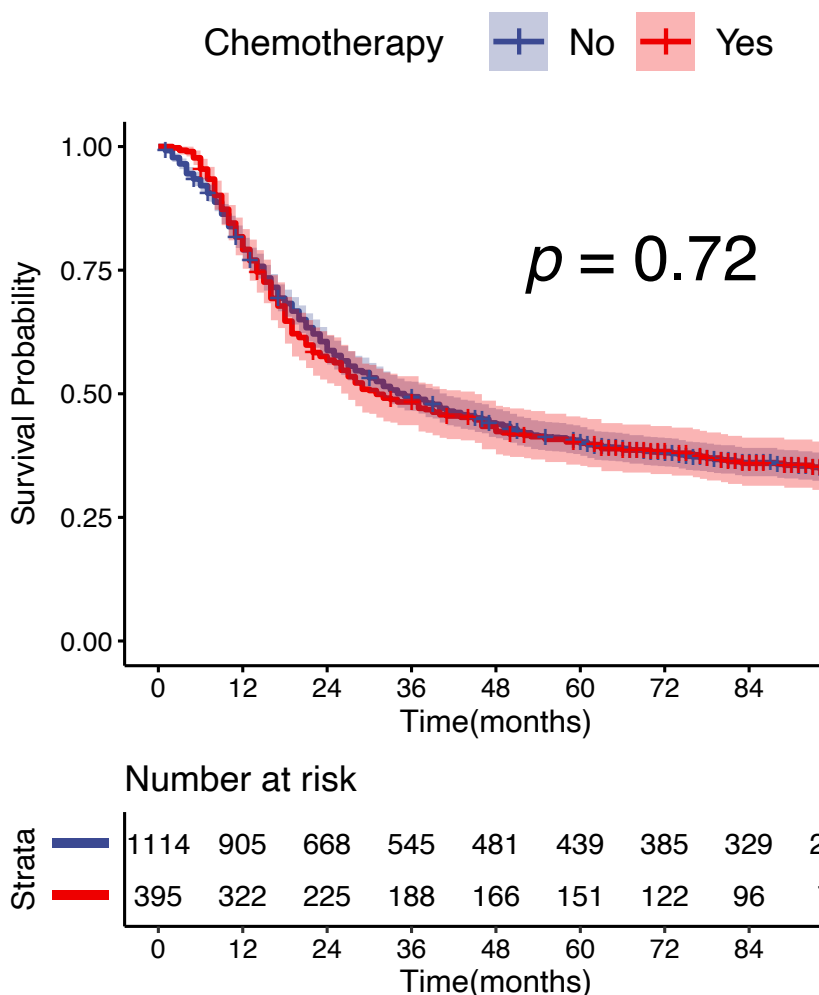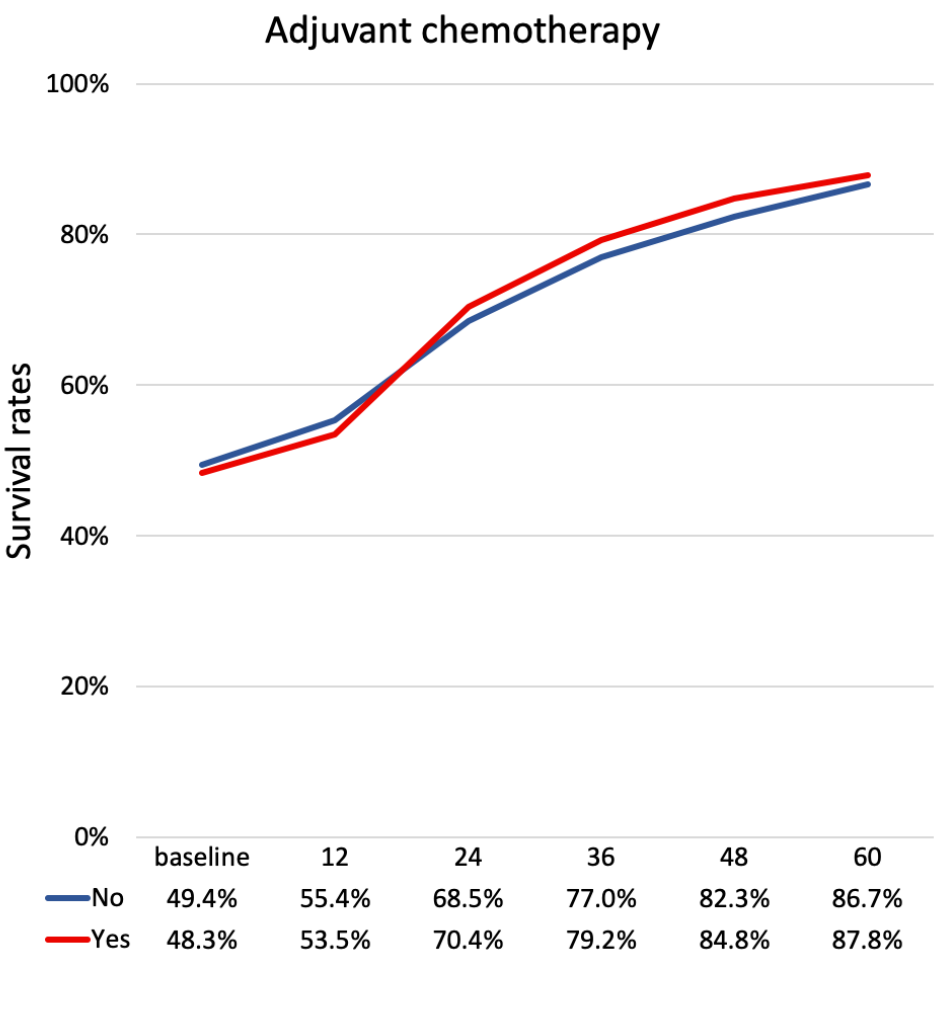

Supplement: Supplementary file 1 — Supplementary file. [file jcav12p1220s1.pdf]
